# Regulation of Isoflavone Biosynthesis by miRNAs in Two Contrasting Soybean Genotypes at Different Seed Developmental Stages

**DOI:** 10.3389/fpls.2017.00567

**Published:** 2017-04-13

**Authors:** Om P. Gupta, Deepti Nigam, Anil Dahuja, Sanjeev Kumar, T. Vinutha, Archana Sachdev, Shelly Praveen

**Affiliations:** ^1^Division of Biochemistry, ICAR-Indian Agricultural Research Institute, Pusa CampusNew Delhi, India; ^2^Centre for Agricultural Bio-Informatics, ICAR-Indian Agricultural Statistics Research Institute, Pusa CampusNew Delhi, India

**Keywords:** miRNA, 4-Coumarate-CoA ligase, daidzein, genistein, glycitein, isoflavone 7-*O*-glucosyltransferase, RA-PCR

## Abstract

Owing to the presence of nutritionally important, health-promoting bioactive compounds, especially isoflavones, soybean has acquired the status of a functional food. miRNAs are tiny riboregulator of gene expression by either decreasing and/or increasing the expression of their corresponding target genes. Despite several works on identification and functional characterization of plant miRNAs, the role of miRNAs in the regulation of isoflavones metabolism is still a virgin field. In the present study, we identified a total of 31 new miRNAs along with their 245 putative target genes from soybean seed-specific ESTs using computational approach. The Kyoto Encyclopedia of Genes and Genomes pathway analyses indicated that miRNA putatively regulates metabolism and genetic information processing. Out of that, a total of 5 miRNAs (*Gma*-miRNA12, *Gma*-miRNA24, *Gma*-miRNA26, *Gma*-miRNA28, and *Gma*-miRNA29) were predicted and validated for their probable role during isoflavone biosynthesis. We also validated their five target genes using RA-PCR, which is as good as 5'RLM-RACE. Temporal regulation [35 days after flowering, 45, 55, and 65 DAF] of miRNAs and their targets showed differential expression schema. Differential expression of *Gma*-miR26 and *Gma*-miRNA28 along with their corresponding target genes (*Glyma.10G197900* and *Glyma.09G127200*) showed a direct relationship with the total isoflavone content. Therefore, understanding the miRNA-based genetic regulation of isoflavone pathway would assist in selection and manipulation to get high-performing soybean genotypes with better isoflavone yield.

## Introduction

Use of soybean has its root in antiquity since the creation of China’s agriculture where this crop was used in medical compilation. Being premier agricultural crops, soybean serves as a major source of vegetable oil (∼20%), protein (∼40%) and animal feed. Global soybean production is recorded as ∼281.9 million metric tons with India having total contribution of 11.9 million tones from an area of ∼12.2 mha, with an average productivity of 9793 hectogram/ha, respectively, (FAO, 2013). It is a rich source of no cholesterol oil (Riaz, 2006) and high quality complete protein (Henkel, 2000). Additionally, soybeans have other vital bioactive compounds such as saponins, protease inhibitors, phytic acid, lecithin and isoflavones (Setchell et al., 2003). Owing to the presence of nutritionally important, health promoting bioactive compounds, especially isoflavones, it has acquired the status of functional food. Its food value in cardiovascular diseases and diabetes is well known (Aerenhouts et al., 2010). The most important isoflavones in soybean is genistein followed by daidzein and glycitein (Wang and Murphy, 1994). In human, isoflavones are reported to have health promoting activity such as lower incidence of cardiovascular disease, hormone-dependent cancers of the breast and prostate (Limer and Speirs, 2004), colon cancer (Rose et al., 1986), menopausal symptoms (Clarkson, 2000), osteoporosis and loss of bone mass intensity (Rochfort and Panozzo, 2007). Due to this tremendous health benefits, they are called “nutraceuticals.” Nevertheless, it also participates in various other biological processes in plants such as antimicrobial phytoalexins, an inducer of nodulation genes during symbiosis, stimulators of fungal spore germination, insect anti-feedants and allochemicals (Ndakidemi and Dakora, 2003).

**FIGURE 1:**
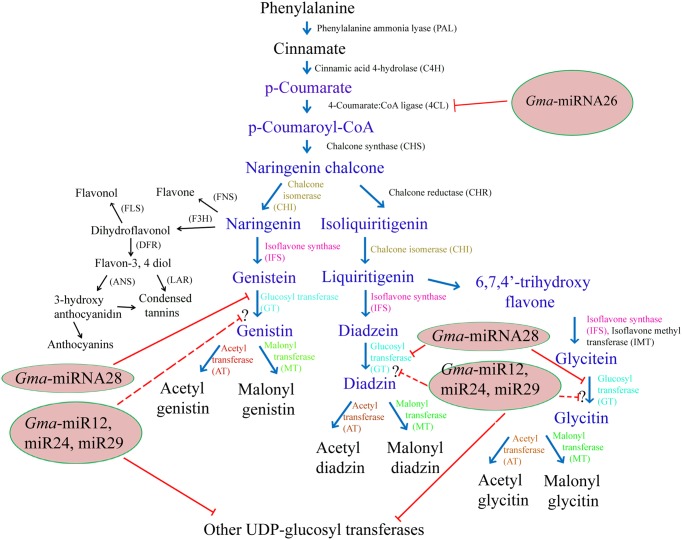
**Schematic representation of probable role of miRNA during isoflavonoid biosynthetic pathway in soybean seed.** Text in blue color represents key metabolites of the isolavonoid pathway. Enzymes targeted by newly identified soybean miRNAs are highlighted by red line. Dotted line represents unclear role of miRNA in the pathway. Reaction catalyzed by the same type of enzyme is shown with same color while other enzymes are mentioned in black color. The competing pathways that share the naringenin and daidzein substrate are in black color. FNS, flavone synthase; F3H, flavone 3-hydroxylase; FLS, flavonol synthase; DFR, dihydroflavonol reductase; ANS, Anthocyanidin synthase; LAR, Flavon-3,4diol 4-reductase.

Isoflavones constitute a distinct group of plant secondary metabolites produced from the phenylpropanoid pathway (**Figure 1**). They are largely produced by members of the Papilionoideae family, including soybean (daidzein, genistein, and glycitein) (Wang and Murphy, 1994). Soybean seeds are a major source of dietary isoflavones. The multistep pathway of the isoflavone biosynthesis starts with the precursor l-phenylalanine, which after non-oxidative deamination forms cinnamic acid *via* the enzyme phenylalanine ammonia lyase (PAL). Then, cinnamic acid is translated into *p*-coumaryol CoA by 4-hydroxylase (C4H) and 4-coumarate CoA ligase (4CL). Chalcone synthase (CHS), a multigenic family in soybean is the first critical enzyme for the isoflavone biosynthesis (Schroder, 2000). CHS7 and CHS8 are seed specific in soybean and catalyzes conversion of p-coumaryol CoA into naringenin chalcone (Dhaubhadel et al., 2007). Chalcone isomerase (CHI) and chalcone reductase (CHR) are other important enzymes required for the isoflavone synthesis (Ralston et al., 2005). 2-hydroxyisoflavanone synthase (isoflavone synthase, IFS) is the crucial enzyme that specifically differentiates isoflavone-producing plants from those with no isoflavone. Isoflavone synthase is a microsomal cytochrome P450 monooxygenase, which catalyzes a 2, 3 aryl ring migration of flavanones to their corresponding flavones and subsequent hydroxylation of the resulting C-2 radical (Akashi et al., 1999; Jung et al., 2000). In soybean genome, two IFS genes (IFS1 and IFS2) differing by 14 amino acids, have been identified (Jung et al., 2000). Both isoforms of IFS convert the naringenin and liquiritigenin flavanones to their corresponding isoflavones (Jung et al., 2000; Dhaubhadel et al., 2003). The difference in the accumulation of isoflavone in different soybean cultivars is the result of interaction of genetic and environmental factors whose regulation is still unclear. Like other plant secondary metabolic pathways, isoflavone biosynthetic pathway is also divided into several branches which generally shares common substrate inviting strong and regulated flux channeling of common substrate. This could be one of the major bottlenecks of metabolic engineering of isoflavone biosynthesis in the plants. Every step of every branch of this pathway is regulated by different genes which decide the accumulation pattern of different compounds of the pathway.

On the other hand, gene expression is tightly regulated both at transcription and post transcription level. Post transcriptional based gene regulation is mediated largely by two classes of small RNA, i.e., miRNA (microRNA) and siRNA (short interfering RNA). Of special interest, miRNAs are 21-24 nucleotides, non-coding, endogenous riboregulator that regulate gene expression in eukaryotes (Khraiwesh et al., 2010). Till date, ∼8465 miRNAs have been deposited from ∼73 plant species in the miRBase (release 21) database^1^. Of those, ∼637 miRNAs belong to soybean which is still growing (Song et al., 2011; Turner et al., 2012; Ye et al., 2014). Owing to such huge number of miRNAs in soybean, works have just started to characterize them for various biological processes such as Cd stress (Fang et al., 2013), cyst nematode-soybean interaction (Li et al., 2012), nodulation (Zhang et al., 2014), Al stress (Zeng et al., 2012), P starvation (Xu et al., 2013), abiotic and biotic stresses (Kulcheski et al., 2011). In addition to soybean, miRNAs are reported for their numerous roles including biotic and abiotic stress, signal transduction (see review Liu and Chen, 2010; Gupta et al., 2014a,b), metabolism, protein degradation (see review Zhang and Wang, 2015) in other plant species.

Nevertheless, very recently, miRNAs have been reported for their involvement in regulating secondary-metabolite biosynthesis in plants (see review Gupta et al., 2017). First evidence of sRNA based secondary metabolism regulation came from Tuteja et al. (2009) where they have shown the tissue specific CHS gene silencing by siRNA in soybean. In Arabidopsis, certain miRNAs such as miR156, miR163, miR393, and miR828 are reported for regulation of secondary metabolites (see review Bulgakov and Avramenko, 2015; Gupta et al., 2017). Nevertheless, miR156-SPL (Squamosa Promoter Binding Protein like) target pair negatively regulates anthocyanin by destabilizing WD40-bHLH-MYB transcription complex (Gou et al., 2011). Additionally, loss of miR163 led to the accumulation of methyl farnesoate (Ng et al., 2011). Very recently, several groups have reported the role of miRNAs in secondary metabolism in various medicinal herbs such as *Taxus mairei, Catharanthus roseus, Papaver somniferum*, and *Picrorhiza kurroa* etc. (Hao et al., 2012; Pani and Mahapatra, 2013; Boke et al., 2015; Vashisht et al., 2015). Despite this information on miRNAs based secondary metabolism regulation, work on the regulatory role of miRNAs during isoflavone biosynthetic pathway in soybean is still a virgin field which requires urgent attention. Keeping in view these facts, we executed experiments and for the first time reporting miRNAs, which target genes of the isoflavone biosynthetic pathway. Moreover, we experimentally validated the temporal expression pattern of miRNAs-target genes pair and also established the relationship between their differential expressions with isoflavone content in two contrasting soybean genotypes (NRC7-low isoflavone and NRC37-high isoflavone).

## Materials and Methods

### *In silico* Identification of miRNAs Targeting Genes of the Isoflavone Biosynthetic Pathway

#### Retrieval of Sequences and Their Pre-processing

For prediction of probable new miRNAs, about 8,465 previously known mature miRNA sequences from whole Viridiplantae belonging to 73 plant species were retrieved from the miRNA Registry database^2^ (Release 21, June 2014). Similarly, about 3,86,859 *Glycine max* ESTs of the reproductive stage of seed development were retrieved from the Unigene nucleotide database available at NCBI^3^. To avoid the overlap, the miRNA data set was screened with the help of an in-house perl script^4^ to remove the redundant miRNA sequences and the remaining sequences were used as a reference miRNA for homologous prediction in *G. max*. All the downloaded EST sequences were subjected to PolyA/T tails trimming using the EST trimmer perl program. Vector sequences and other contaminations were identified by using VecScreen web server^5^. Low complexity regions were masked by using Repeat Masker^6^. CAP3 software was used to assemble the trimmed EST sequences into the contigs and singlets.

#### Prediction of miRNAs, Their Secondary Structure and Phylogenetic Analysis

**FIGURE 2:**
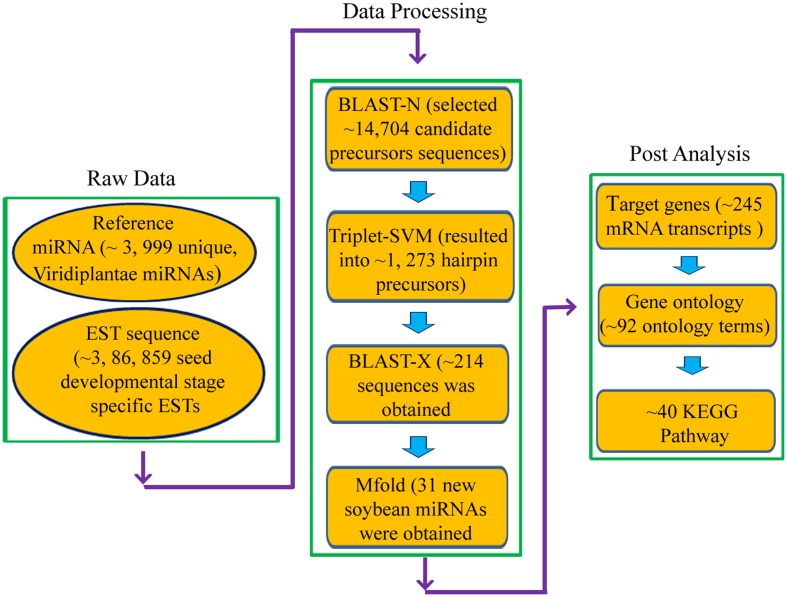
**Schematic workflow for prediction of miRNAs and their targets in Soybean**.

Schematic representation of miRNA prediction pipeline is given in **Figure 2**. The reference miRNA sequences were used as query sequences for BLASTN search against the soybean EST database locally. Only unique miRNA was aligned pairwise by BLASTN program with a threshold (*e*-value at 10) and the word-match size between the query and the database was kept at 7 bp. The potential new miRNAs were selected if they had a length of 18 nucleotides (ntd) with no gaps and mismatch not more than 3 between query miRNA and aligned ESTs. Wherever available, the precursor sequence of 50 ntd upstream and downstream to the BLAST hits were extracted and used for hairpin structure prediction. To predict real miRNA precursor, triplet-SVM classifier program (Xue et al., 2005) was used. Filtered precursors were subjected to BLASTx and Rfam search to remove protein coding sequences and to distinguish between miRNA and other RNA families such as rRNA, snRNA, tRNA, respectively (Griffiths-Jones et al., 2005).

RNAfold algorithm was employed to generate promising stable secondary structures from the precursors. The parameters used for the secondary structure prediction were: minimum free energy and partition function; avoid isolated base pairs; dangling energy on both sides of the helix in any case; RNA parameters; rescale energy parameters at a given temperature 37°C; interactive RNA secondary structure plot; RNA secondary structure plots with reliability annotation and mountain plot (Turner and Mathews, 2009). Additionally, these secondary structures were manually curated following rules of Zhang et al. (2005). The precursor and mature sequences of the identified new miRNAs of isoflavonoids pathway were subjected to phylogenetic analysis to explore their evolutionary relationships^7^. Evolutionary distances were calculated neighbor-joining (NJ) method 49 following 1000 bootstrapped replicates. All the analyses were performed using the MEGA v4.0 software.50.

#### Prediction of Target Genes and Their Functional Characterization

To predict the potential target genes of the identified miRNAs, the draft genome sequence of *G. max* was downloaded from PlantGDB database^8^. These genome sequences were then used for prediction of miRNA targets using psRNATarget web server^9^ (Dai and Zhao, 2011) following parameters mentioned by Nigam et al. (2015). To better understand the biological function of predicted target genes of newly identified miRNAs, AgriGO tool^10^ under the gene ontology system (Du et al., 2010) was employed. The pathways and the network of molecular interaction of the predicted target genes were studied by KEGG^11^. For enrichment analysis, a hypergeometric distribution based statistical test (level of significance at 0.05%) was used to reject the chances of randomness in association to target genes with their corresponding ontology term. To moderate the false positives in multiple hypotheses testing procedure Benjamini and Hochberg false discovery rate (FDR) correction was applied (Benjamini and Hochberg, 1995).

### *In planta* Validation of miRNAs Targeting Genes of Isoflavone Biosynthetic Pathway

#### Plant Materials, Growth Conditions, and Sample Preparation

In the present study, we selected two extreme contrasting soybean genotypes (NRC7 and NRC37) differing in the total isoflavone content. The genotype NRC7 is a selection from S69-96 while genotype NRC37 is a cross of Gaurav X Pb1 (selection from Nanking) and contains 490.4 and 1634.5 μg/g isoflavone content, respectively (Kumar et al., 2010). Both the genotypes were procured from ICAR-Directorate of Soybean research, Indore, India. Prior to sowing, the seeds were initially treated with 1% sodium hypochlorite for 10 min, rinsed in distilled water for three times. Sterilized soybean seeds were sown in 6^″^ pot filled with autoclaved standard agro-coir peat growing media (Shaa Pith Media Company, India) in a 15 square feet growth chamber maintained at ICAR-National Phytotron Facility, Pusa Campus, IARI, New Delhi-110012. After a light sprinkling of water, pots were shifted to the growth chamber and maintained up to maturity with a growing condition of day/night temperature of 28/26°C, relative humidity ∼75% with a 16 h light duration. For miRNAs and their target validation, we selected four stages of soybean seed development which maximally contribute to the isoflavone accumulation, i.e., 35 days after flowering (DAF), 45, 55, and 65 DAF (**Supplementary Figure S1A**). Each stage of each genotype was replicated three times. Seed samples were harvested at above mentioned stages and immediately frozen into liquid nitrogen and stored at −80°C till further use.

#### Small RNA Isolation and Small RNA cDNA Library (srcDNA) Construction

Total small RNA was isolated from 100 mg of seed tissue using the mirVanaTM isolation Kit (Ambion) following manufacture’s instruction. Small RNA concentration was checked using Nano-Drop (NanoDrop technologies). For validation of isoflavone pathway related miRNAs, srcDNA library was constructed following poly (T) RTQ primer (**Supplementary Table S1**) based protocol described earlier (Ro et al., 2006; Gupta et al., 2012). Briefly, isolated miRNAs were exposed to Poly (A) tailing at 3′ end of miRNA using poly(A) polymerase enzyme (NEB) at 37°C for 45 min. After tailing, samples were purified using mirVana Probe and Marker Kit (Ambion). Poly(A) tailed miRNAs were reverse transcribed using RTQ primer (**Supplementary Table S1**) by PrimeScript^TM^ 1st strand cDNA Synthesis Kit (TaKaRa, Japan) at 42°C for 60 min. and 5 U RNase H (NEB) was added to remove small RNAs. The srcDNA concentration was quantified using a NanoDrop spectrophotometer (NanoDrop technologies).

#### Quantitative Real Time PCR of Newly Identified miRNAs

Amongst all the newly identified miRNAs, five miRNAs (*Gma*-miR12, *Gma*-miR24, *Gma*-miR26, *Gma*-miR28, and *Gma*-miR29) which are predicted to be involved in isoflavone pathway were selected for qRT-PCR based validation at the different seed developmental stage. Expression of miRNAs was carried out using miRNA specific forward primer and universal reverse primer (**Supplementary Table S1**). We selected three reference genes as internal control primer, i.e., 5s rRNA, miR172ab and miR1520d (**Supplementary Table S1**) for data normalization. All the primers were validated using thermal dissociation (Tm) curve of PCR amplicons which showed single peak.

Amplification of each miRNA was established with PCR following conditions of 94°C for 5 min, then 40 cycles of 94°C for 15 s, 55°C for 30 s and 72°C for 45 s. PCR products were resolved using 3% agarose gel electrophoresis with a size of ∼120 bp (**Supplementary Figure S1B**). qPCR was carried out using Bio-Rad CFX96 machine in a reaction volume of 20 μl containing SYBR^^®^^ Green JumpStart^TM^ Taq ReadyMix^TM^ (Si*Gma*), RTQ-UNIr primer (10 μM), forward primer (10 μM) and 10 ng/μl of srcDNA using above mentioned PCR protocol. The relative expression level was calculated following 2^-ΔΔCt^ method (Livak and Schmittgen, 2001). Standard errors and standard deviations were calculated from replicates of three biological tissues.

#### *In vivo* Validation of Target Transcripts Using Regional Amplification Quantitative RT-PCR (RA-PCR)

**FIGURE 3:**
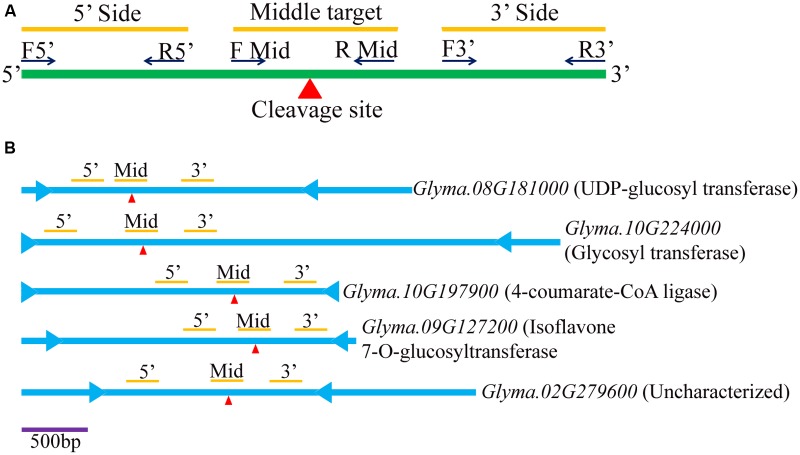
**Diagram of regional amplification quantitative RT-PCRs (RA-PCR). (A)** Diagram of primers designed to amplify fragments of cleaved and non-cleaved miRNA (miRNA)-targeted mRNA present in tissues. **(B)** Five target genes (*viz.* *Glyma.08G181000, Glyma.10G224000, Glyma.10G197900, Glyma.09G127200*, and *Glyma.02G279600*) are illustrated for relative locations of start (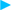) and stop (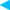) codons, miRNA target sites (red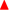), and three bars representing 5′, middle target and 3′ regions (from left to right).

To further measure and validate the expression levels of the predicted target transcripts of the isoflavone pathway related soybean miRNA, regional amplification quantitative RT-PCR (RA-PCR) assay was performed. Total 5 target transcripts were used (**Supplementary Table S2**). The RA-PCR was developed to monitor the miRNA-directed cleavage of mRNAs (Oh et al., 2008). MicroRNA mediated cleavage of target site of mRNA transcripts lead to decrease in RT-PCR accumulation of any fragment present upstream of the target site (Navarro et al., 2006). The reverse transcription of the miRNA-cleaved mRNA will not generate a cDNA beyond the cleaved site. Therefore, the segment of cDNA present upstream of cleaved site can be expected to be less abundant than a segment present downstream of the cleaved site. RA-PCR has added benefits of including control reaction in the same experiment while other technique such as 5′ rapid amplification of cDNA ends (RACE) 5′ RACE requires separate reaction and could be skewed by the presence of false positives. For each potential mRNA target transcripts, a total of three sets of primers were designed to amplify three different regions (5′ region, middle region and 3′ region) (**Figure 3**; **Supplementary Table S2**). Total RNA was isolated from 100 mg seed tissue from four stages of soybean seed development mentioned above in the material section using Trizol (Invitrogen) following user’s manual. Other steps of cDNA synthesis, primer validation (**Supplementary Figure S1C**) and qRT-PCR were practiced same as described for miRNA studies.

### HPLC Analysis of Isoflavone Content in Contrasting Soybean Genotypes Temporally

#### Sample Preparation of Extracts

Soybean seeds were harvested at different stages of seed development from two contrasting soybean genotypes mentioned in plant material section. Three biological replications were used throughout the experiment. Sample preparation of extracts was performed following standard procedure of Vyn et al. (2002) and Kumar et al. (2010). Briefly, Seeds were completely dried in hot oven at 70°C to remove the available moisture. Dried seeds were finely ground and filtered by passing through 100-mesh sieve. About 125 mg of finely ground soy flour was mixed with 5 ml of 80% ethanol and 1ml of concentrated HCl and extracted for 2 h in a boiling water bath. This method of sample extraction, largely depends on acid hydrolysis of 12 different endogenous isomers to their corresponding aglycone forms such as daidzein, glycitein, and genistein. The resulting suspension after extraction was centrifuged at 10000 rpm for 10 min to get the clear supernatant.

#### HPLC Conditions and Standard Curve Preparation

Before injecting the sample into the HPLC instrument, the supernatant obtained after centrifugation filtered using 0.5 micron syringe filter having diameter 25 mm. Twenty microliter of the syringe-fltered sample was injected into a Waters 2695 chromatograph (Waters Corp., Milford, MA, USA), equipped with a 2998 photodiode array detector (Waters Corp., Milford, MA, USA) housing a C-18 silica column (Phenomenex; 5 l with dimension of 250 × 4.6 mm). The separation and elution of isoflavones were accomplished by employing a binary gradient mode with solvent A (water with 0.1% formic acid) and solvent B (Acetonitrile with 0.1% formic acid) at a flow rate of 1.25 ml/min for 25 min. The solvent system was run as follows (% solvent A/solvent B): 0 min (87/13), 1 min (87/13), 20 min (70/13), 25 min (87/13). The resolution of isoflavones as detected at 260 nm is shown in chromatogram (**Supplementary Figure S2**)

The standard stock solution (500 ppm) of daidzein, glycitein, and genistein (Sigma) was prepared by dissolving 5 mg of these compounds into 10 ml of 80% ethanol. Using this stock solution, further dilutions were prepared to get five point linear calibration curve containing 5, 10, 20, 40, and 50 ppm of these compounds. The calibration curves were obtained by plotting peak area versus concentration. The relative concentration of each isoflavone in the test samples was obtained from software CSW version 1.7 after superimposing the chromatogram of the test sample on the standard curve and was expressed as μg/g on dry weight basis. Furthermore, concentrations of all the aglyconic forms were summed up to obtain total isoflavone contents.

## Results

### *In silico* Identification and Characterization of miRNAs and Their Targets Involved in the Isoflavone Biosynthetic Pathway

#### Identification of miRNAs

To discover new miRNAs related to isoflavone biosynthetic pathway in soybean, we exploited information of 8, 465 mature miRNAs from Viridiplantae submitted in miRBase database and 3, 86, 859 seed developmental stage specific ESTs from NCBI. The total ESTs were pre-processed to remove poly-A/T tail followed by assembly into 41, 235 contigs and 312, 211 singlets. Now, these assembled ESTs sequences were subjected for homolog search against 3, 999 unique, Viridiplantae miRNAs. Using in-house Perl script, we could select 14,704 candidate precursors sequence with a flanking region of 50 nucleotides. As a thumb rule, precursors must form a hairpin structure; all these precursors were further processed with Triplet-SVM which resulted in 1273 hairpin precursors. Using RNAfold algorithm, stable secondary structure of 214 sequences was obtained after discarding the ESTs having similarity with non-redundant protein database. Following Zhang et al. (2005), precursors were manually curated and a 31 new miRNAs were obtained (**Supplementary File S1**). Amongst all, based on their target genes function, 5 miRNAs (*Gma*_miRNA12, *Gma*_miRNA24, *Gma*_miRNA26, *Gma*_miRNA28, and *Gma*_miRNA29) were found to be involved in the aforementioned pathway (**Table 1** and **Supplementary File S1**) and their secondary structure is given in **Supplementary Figure S3**.

**Table 1:** Newly identified five miRNAs of isoflavone biosynthetic pathway from Soybean ESTs.

Sr. No.	miRNA header	Mature miRNA	Mature length	Precursor length	GC%	MFE (kcal/mol)	MFEI	AMFEI	AU%	Homologs miRNAs
(1)	>*Gma*_miRNA12	AGACAGUUAUUUUGGGACGGA	21	121	41.32231	-44.0	-0.88	-36.36	58.67769	hvu-miR5049c
(2)	> *Gma* _miRNA24	UCUUGAAGUCUCGCUUGCAG	20	120	49.16667	-36.3	-0.61	-30.25	50.83333	bdi-miR7741-5p.2
(3)	> *Gma* _miRNA26	UAAUUGUCGCAGUUUUGAACU	21	121	31.40496	-36.1	-0.95	-29.83	68.59504	bdi-miR5180b
(4)	> *Gma* _miRNA28	UCUGUACCAUAAUAUAAGAC	20	120	40	-35.1	-0.73	-29.25	60	huv-miR1130
(5)	> *Gma* _miRNA29	UAGAUACAUCCAUAUGUAGA	20	120	35	-34.4	-0.81	-35.66	65	tae-miR1122a


#### Identification of Target Genes

**FIGURE 4:**
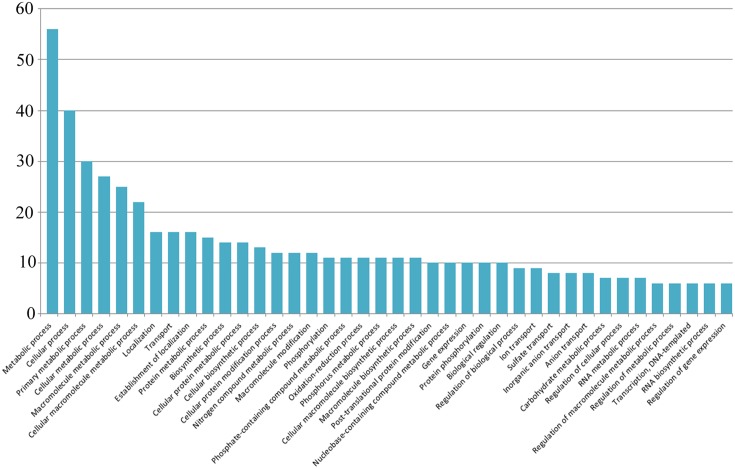
**Kyoto Encyclopedia of Genes and Genomes (KEGG) pathway results.** The *y*-axis represents the abundance of the pathways.

In this study, a total of 245 mRNA transcripts putatively targeted by 31 newly identified soybean miRNAs were predicted (**Supplementary File S2**). For a more comprehensive annotation, both the Gene Ontology (GO) and the Kyoto Encyclopedia of Genes and Genomes (KEGG) pathway analyses were performed (**Supplementary File S3**). Multiple genes were found to be targeted by a single miRNA. For instance, *Gma*_miRNA4 is predicted to target 23 transcripts which are involved in diverse biological processes. Nevertheless, *Gma*_miRNA11 was found to have maximum targets (23) while *Gma*_miRNA1 had lowest target (1). As the ESTs for miRNA prediction were collected from different seed developmental stages, most of the target genes were expected to be involved in various metabolic processes. A total of 92 ontology terms representing 40 KEGG pathways were observed, with most of the target genes being involved in response to metabolic process followed by localization, transport and biosynthetic process *etc.* (**Figure 4**). Although most of the target genes were predicted to be involved in metabolic processes, but unfortunately, we could identify only five target genes (*Glyma.08G181000, Glyma.10G224000, Glyma.10G197900, Glyma.09G127200*, and *Glyma.02G279600*) involved in the isoflavone biosynthetic pathway (**Table 2**).

**Table 2:** List of target genes of five new miRNAs of isoflavonoid pathway.

Sr. No.	New miRNA	Target Id	Target Name_Predicted	Target function_Predicted	Gene Ontology Biological Process_Predicted	Gene Ontology Cellular Component Descriptions_Predicted
(1)	*Gma* _miRNA12	Glyma.08G181000	UDP-glucoronosyl and UDP-glucosyltransferase	Transferase activity, transferring glycosyl and hexosyl groups	Metabolic process	Cellular component
(2)	*Gma* _miRNA24	Glyma.10G224000	UDP-glucoronosyl and UDP-glucosyltransferase, glycosyltransferase family 28 N-terminal domain	Sterol 3-beta-glucosyltransferase activity, transferring glycosyl and hexosyl groups	Carbohydrate metabolism, flavonoid biosynthesis, lipid glycosylation	Nucleus, vacuolar membrane, vacuole
(3) .	*Gma* _miRNA26	Glyma.10G197900	4-coumarate-CoA ligase	AMP-binding enzyme	JA biosynthesis phenylpropanoid metabolism, response to chitin, fungus, JA stimulus and wounding	Chloroplast, peroxisome
(4)	*Gma* _miRNA28	Glyma.09G127200	Isoflavone 7-*O*-glucosyltransferase 1-like (based on Primer blast result)	UDP-glycosyltransferase activity, quercetin 3′-*O*-, 4′-*O*-,7-*O*-glucosyltransferase activity	Metabolic process	Cytosol, nucleus
(5)	*Gma* _miRNA29	Glyma.02G279600	Uncharacterized	Transferase activity, transferring glycosyl groups	Biological process	Golgi apparatus, endosome, *trans*-Golgi network


#### Sequence Alignment and Phylogenetic Analysis of the New miRNAs

Precursor and mature miRNAs are highly conserved among distantly related plant species. Amongst 31 newly identified soybean miRNAs, five isoflavone pathway related mature miRNAs (*Gma*_miRNA12, *Gma*_miRNA24, *Gma*_miRNA26, *Gma*_miRNA28, and *Gma*_miRNA29) were used to find out the best closely related miRNAs in other plant species. *Gma*_miRNA12 was best closely related to *huv*-miR5049c among 15 miRNA homolog in different species (**Supplementary Figure S4A**) while *Gma*_miRNA24 had only one homolog, i.e., *bdi*-miR7741-5p.2. Similarly, *Gma*_miRNA26 was best closely related to *bdi*-miR5180b (**Supplementary Figure S4B**) while *Gma*_miRNA28 showed a close relation with *huv*-miR1130 (**Supplementary Figure S4C**). In addition, *Gma*_miRNA29 showed homology with *tae*-miR1122a (**Supplementary Figure S4D**).

### Validation and Expression Profiling of the Isoflavone Biosynthetic Pathway Related miRNAs and Their Targets in Contrasting Soybean Genotypes Temporally

A total of 31 new miRNAs were mined using computational approach from soybean seed ESTs. Amongst all, five miRNAs (*Gma*_miRNA12, *Gma*_miRNA24, *Gma*_miRNA26, *Gma*_miRNA28, and *Gma*_miRNA29) were targeting the genes (*Glyma.08G181000, Glyma.10G224000, Glyma.10G197900, Glyma.09G127200*, and *Glyma.02G279600*) of the isoflavone biosynthetic pathway. Quantitative PCR and RA-PCR was employed for experimental validation of these miRNAs and their targets, respectively, at four seed developmental stages (35, 45, 55, and 65 DAF) in contrasting soybean genotypes. The amplified products were resolved on 2% agarose gel and amplicons of respective miRNAs and their target mRNAs were detected.

#### Expression Profiling of miRNAs and Their Targets in NRC7 Genotype

**FIGURE 5:**
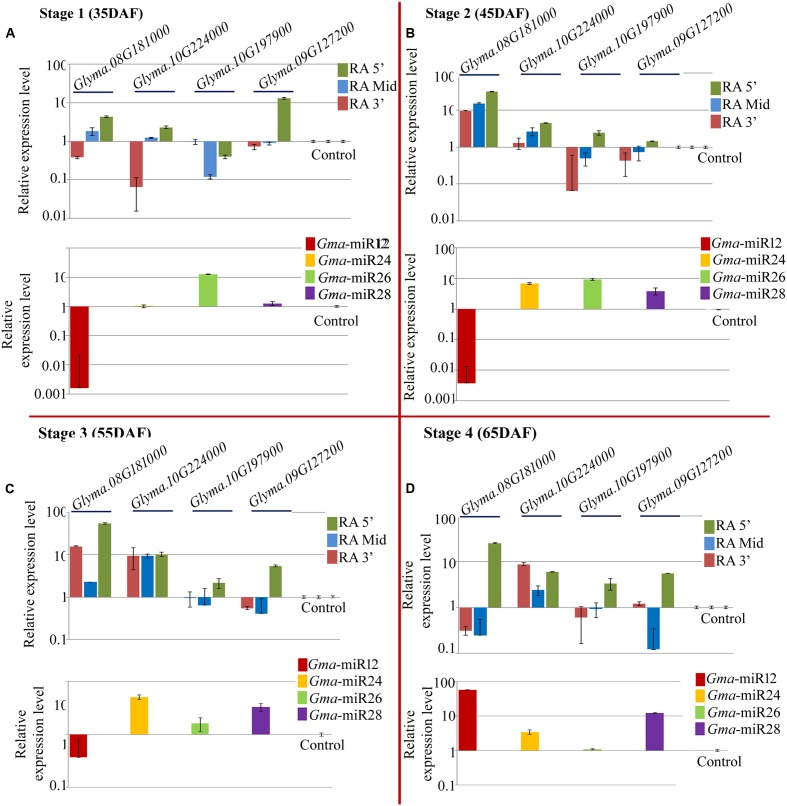
**Relative expression level of newly identified target genes (UDP-glucosyltransferase: *Glyma.08G181000*; UDP-glucosyltransferase: *Glyma.10G224000*; 4-coumarate-CoA ligase: *Glyma.10G197900*; isoflavone 7-*O*-glucosyltransferase: *Glyma.09G127200*) along with their corresponding miRNAs (*Gma*-miR12, *Gma*-miR24, *Gma*-miR26, and *Gma*-miR28) in NRC7 soybean cultivar at four developmental stages, i.e., (A)** 35 DAF; **(B)** 45 DAF; **(C)** 55 DAF, and **(D)** 65 DAF. Control refers to *Glyma.02G279600* and *Gma*-miR29 for target and miRNA, respectively, which was used to calculate ΔΔCt value throughout all the stages. Three reference genes (EF 1α 2a, Cyclophillin and Actin 2/7) were used to calculate accurate normalized value using Genorm software. The expression level of miRNAs as well as its target gene was normalized to the normalized value of reference genes on a logarithmic scale. Error bar represents ± SE of three independent biological replications.

Quantitative PCR was performed for the expression analysis of the newly identified mature miRNAs and their target mRNAs in NRC7 genotype (**Figure 5** lower panel). *Gma*-miRNA24, *Gma*-miRNA26, and *Gma*-miRNA28 showed up regulation (**Figures 5B–D**) among all the four stages with varying level of accumulation while one miRNA *Gma*_miRNA12 showed differential expression landscape (**Figure 5A** lower panel). Among all the miRNAs and stages studied, *Gma*_miRNA12 showed maximum accumulation (∼58.82-fold) at 65 DAF while down regulation at 35, 45, and 55 DAF (**Figure 5A** lower panel). Two miRNAs such as *Gma*_miRNA24, *Gma*_miRNA26 showed maximum accumulation of ∼6.9 and ∼9.3-fold at 45 DAF, respectively (**Figures 5B,C** lower panel), while *Gma*_miRNA28 showed (∼12.29-fold) at 65 DAF (**Figure 5D** lower panel).

Quantitative RT-PCR was employed to assess the abundance of three different regions of five target genes (*Glyma.08G181000, Glyma.10G224000, Glyma.10G197900, Glyma.09G127200*, and *Glyma.02G279600)* targeted by five newly identified miRNAs. **Figure 3A** depicts the relative positions of the three primer sets used for a given target transcript for the RA-PCR method. The first set (F5′, R5′), second set (F Mid, R Mid) and third set (F3′, R3′) amplifies upstream, target site and downstream site from the potential cleavage site, respectively. As expected, the expression result of all the miRNAs except *Gma*-miRNA24, were negatively correlated with their target transcripts at least for RA-Mid region of the transcripts at all the stages of seed development (**Figure 5** upper panel). The accumulation of RA-Mid region of all the transcripts (*Glyma.08G181000, Glyma.10G224000, Glyma.10G197900, Glyma.09G127200*, and *Glyma.02G279600)* was found to be lower than the RA 3′ region at all the stages of seed development (**Figure 5** upper panel) supporting the fact that these target genes are cleaved by corresponding miRNAs (*Gma*_miRNA12, *Gma*_miRNA24, *Gma*_miRNA26, *Gma*_miRNA28, and *Gma*_miRNA29). The reduction in the accumulation of RA-Mid region compared to RA 3′ region of *Glyma.08G181000, Glyma.10G224000, Glyma.10G197900*, and *Glyma.09G127200* was varying from 51.9 to 99.06%, 8.8 to 61.6%, 61.9 to 83.3%, and 64.2 to 98.8%, respectively, across all the stages. Additionally, compared to target site and RA 3′ region of all the transcripts, the accumulation pattern of RA 5′ region was highly differential suggesting the differential stability index.

#### Expression Profiling of miRNAs and Their Targets in NRC37 Genotype

**FIGURE 6:**
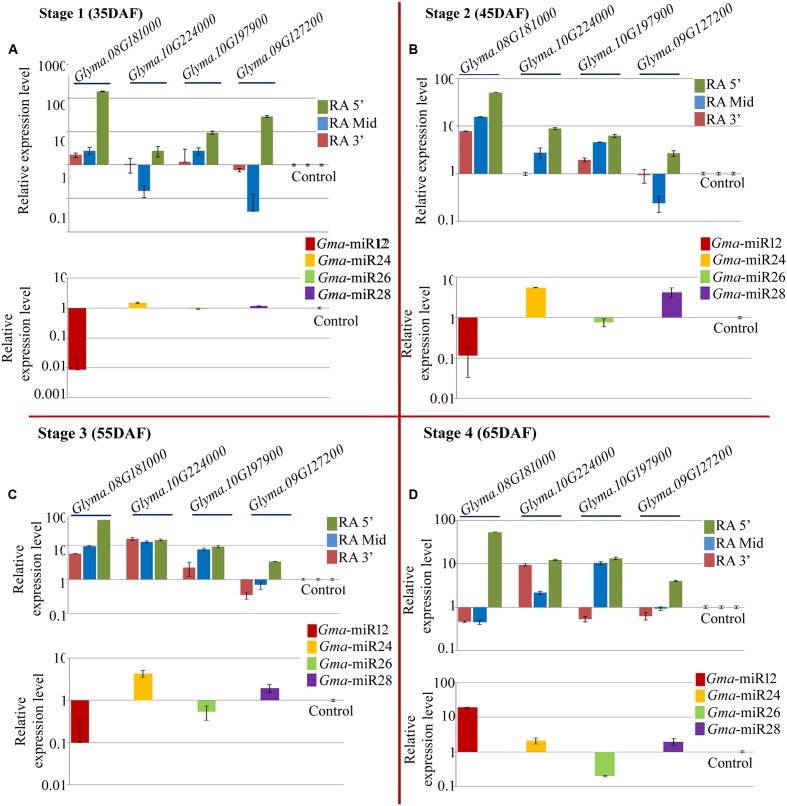
**Relative expression level of newly identified target genes (UDP-glucosyltransferase: *Glyma.08G181000*; UDP-glucosyltransferase: *Glyma.10G224000*; 4-coumarate-CoA ligase: *Glyma.10G197900*; isoflavone 7-*O*-glucosyltransferase: *Glyma.09G127200*) along with their corresponding miRNAs (*Gma*-miR12, *Gma*-miR24, *Gma*-miR26, and *Gma*-miR28) in NRC37 soybean cultivar at four developmental stages, i.e., (A)** 35 DAF; **(B)** 45 DAF; **(C)** 55 DAF, and **(D)** 65 DAF. Control refers to *Glyma.02G279600* and *Gma*-miR29 for target and miRNA, respectively, which was used to calculate ΔΔCt value throughout all the stages. Three reference genes (EF 1α 2a, Cyclophillin and Actin 2/7) were used to calculate accurate normalized value using Genorm software. The expression level of miRNAs as well as its target gene was normalized to the normalized value of reference genes on a logarithmic scale. Error bar represents ± SE of three independent biological replications.

As in case of NRC7, quantitative PCR was performed for the expression analysis of the newly identified mature miRNAs and their target mRNAs in NRC37 genotype (**Figure 6** lower panel). All the studied miRNAs showed differential expression behavior at all the stages of seed development (**Figure 6** lower panel). Interestingly, like NRC7 genotype, *Gma*_miRNA12 showed similar trends of accumulation in NRC37 with down regulation at 35, 45, and 55 DAF while up regulation at 65 DAF (**Figure 6A** lower panel). Two miRNAs namely *Gma*_miRNA24 and *Gma*_miRNA28 showed up regulation at all the stages of seed development in NRC37 genotypes (**Figures 6B,D** lower panel). In contrast, one miRNA, i.e., *Gma*_miRNA26 showed down regulation at all the stages under study (**Figure 6C** lower panel). Unlike NRC7 genotype, maximum accumulation of *Gma*_miRNA12, *Gma*_miRNA24 and *Gma*_miRNA28 was reported at 65 DAF (∼19.22-fold), 45 DAF (∼5.5-fold), and 45 DAF (∼4.2-fold) respectively (**Figures 6A,B,D**).

Similarly, quantitative RT-PCR was employed to assess the abundance of three different regions of above mentioned target genes. Here also, the expression result of all the miRNAs was negatively correlated with their target transcripts at least for RA-Mid region of the transcripts at four different stages of seed development (**Figure 6** upper panel). The accumulation of RA-Mid region of all the transcripts (*Glyma.08G181000, Glyma.10G224000, Glyma.10G197900, Glyma.09G127200*, and *Glyma.02G279600*) was found to be lower than the RA 3′ region at all the stages of seed development (**Figure 6** upper panel) supporting the fact that these target genes are cleaved by corresponding miRNAs (*Gma*_miRNA12, *Gma*_miRNA24, *Gma*_miRNA26, *Gma*_miRNA28, and *Gma*_miRNA29). The reduction in the accumulation of RA-Mid region compared to RA 3′ region of *Glyma.08G181000, Glyma.10G224000, Glyma.10G197900*, *Glyma.09G127200* was varying from 69.3 to 99.16%, 13.1 to 96.1%, 17.2 to 29.1%, and 88.4 to 97.8%, respectively, across all the stages. Additionally, compared to target site and RA 3′ region of all the transcripts, the accumulation pattern of RA 5′ region was highly differential suggesting the differential stability index.

### Accumulation Pattern of Total Isoflavones in Two Contrasting Soybean Genotypes at Different Seed Developmental Stages

**FIGURE 7:**
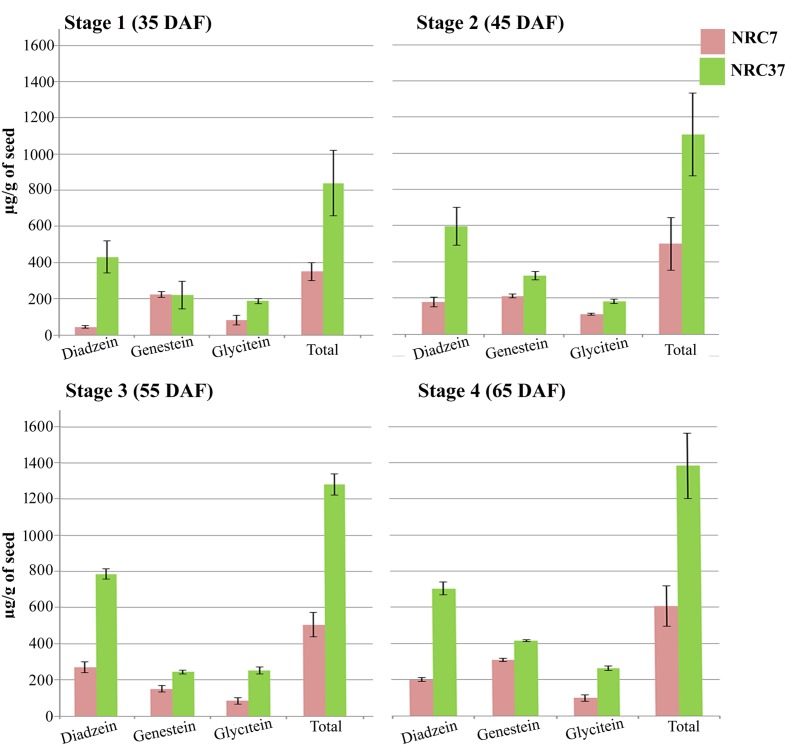
**Differential accumulation pattern of aglycone form of individual and total isoflavones during various stages of seed development in two contrasting soybean genotypes.** Data are the means of measurements from three independent biological replicates. Bars represent the SE.

Using HPLC, the accumulation pattern of different aglyconic forms individually as well as total isoflavones was monitored in NRC7 and NRC37 soybean genotypes under four seed developmental stages (35, 45, 55, and 65 DAF). Our results indicated compositional variation in aglyconic forms of isoflavones across all the developmental stages (**Figure 7** and **Supplementary Table S3**). Increasing trend of accumulation of total isoflavones was observed across all the stages (**Figure 7**) with a maximum accumulation of 606.36 and 1382.51 μg/g in NRC7 and NRC37, respectively, at 65 DAF. Interestingly, the trend of accumulation of daidzein was higher followed by genistein and glycitein in all the stages in NRC37 genotype while same was differential in NRC7 genotype (**Figure 7**).

## Discussion

Soybean isoflavones has attained the status of nutraceuticals in human. In plants, they play a crucial role in various biological processes such as antimicrobial phytoalexins, an inducer of nodulation genes during symbiosis, stimulators of fungal spore germination, insect anti-feedants and allochemicals (Ndakidemi and Dakora, 2003). For decades, miRNAs have been reported as the potent regulatory molecule of various biotic and abiotic stresses, plant growth and development. Recently, these tiny ribonucleotides have emerged as a key regulator of plant secondary metabolites (see review Gupta et al., 2017). As discussed in introduction section that despite several works on miRNAs in soybean, regulation of isoflavone accumulation by miRNAs in soybean is still a virgin field. Owing to the importance of the isoflavone, we executed a three step experiment, i.e., (1) mining of new miRNAs and their target genes from seed specific soybean ESTs that could be involved in regulating the isoflavone biosynthetic pathway; (2) experimental validation of newly identified miRNAs and their target genes; (3) correlation of expression landscape of miRNAs and their targets with isoflavone accumulation in two contrasting soybean genotypes (NRC7: low isoflavone; NRC37: high isoflavone) at four seed developmental stages (35, 45, 55, and 65 DAF).

Recently, Computational identification of miRNAs using ESTs has been a decent strategy for identifying new miRNAs in many crops such as wheat (Pandey et al., 2013), coffee (Akter et al., 2014), *Vigna unguiculata* (Lu and Yang, 2010), *C. roseus* (Pani and Mahapatra, 2013) etc. However, there is only one report stating computation identification of miRNAs in soybean (Guo et al., 2014). Despite several attempts to identify novel and new soybean miRNAs, information on the regulatory role of miRNAs along with their targets in the isoflavone biosynthesises pathway is absent. Keeping in view the importance of ESTs based miRNAs identification; we computationally identified 31 new soybean miRNAs along with their 245 target genes from seed specific soybean ESTs. Owing to the fact that most of isoflavone biosynthesis and accumulation occurs in the developing seeds, we collected ESTs from developing soybean seed to maximize the chance of getting potential new miRNAs related to the isoflavonid pathway. Out of these 31 new miRNAs and their 245 corresponding targets, we could find only five miRNAs and their five target genes that were predicted to be involved in isoflavone pathway regulation (**Figure 1** and **Table 2**). The homologous miRNAs corresponding to these five new miRNAs are given in **Table 1**. All the predicted miRNAs were strictly following the criteria consistent with the previous reports (Zhang et al., 2006). The predicted target genes were normally involved in response to metabolic process followed by localization, transport and biosynthetic process etc. (**Figure 4**). As reported by previous workers (Eldem et al., 2012; Yanik et al., 2013), more than one target genes were predicted to be targeted by a single newly identified soybean miRNAs. Metabolic and cellular processes were primary pathways regulated by newly mined soybean miRNA. Due to their post-transcriptional regulatory roles, it is highly expected that target genes of the miRNA were involved in several genetic-information processing mechanisms. GO analyses revealed that the target genes of newly mined soybean miRNA are mostly involved in response mechanisms, which is in agreement with previous work (Gurkok et al., 2014).

In addition, we used qPCR and RA-PCR to validate and check the expression landscape of newly identified miRNAs (*Gma*_miRNA12, *Gma*_miRNA24, *Gma*_miRNA26, *Gma*_miRNA28, and *Gma*_miRNA29) and their target genes (*Glyma.08G181000, Glyma.10G224000, Glyma.10G197900, Glyma.09G127200*, and *Glyma.02G279600*) respectively, at four different seed developmental stages. Very recently, the RA-PCR was developed to monitor the miRNA-directed cleavage of mRNAs (Oh et al., 2008). MicroRNA mediated cleavage of target site of mRNA transcripts leads to decrease in RT-PCR accumulation of any fragment present upstream of the target site (Navarro et al., 2006). Therefore, the segment of cDNA present upstream of cleaved site can be expected to be less abundant than a segment present downstream of the cleaved site. Our results have indicated higher accumulation of 3′region of about all the target transcripts compared to the middle region signifying the potential cleavage by the corresponding miRNAs.

All the studied miRNAs and their target genes showed differential expression schema across all the stages of seed development except stage 4 (65 DAF) where all the miRNAs showed up regulation in both the soybean genotypes (NRC7 and NRC37). Interestingly, amongst all the miRNA-target pair, expression of *Gma*-miRNA26 and *Gma*-miRNA28 and their corresponding targets *Glyma.10G197900* (4-coumarate-CoA ligase) and *Glyma.09G127200* (isoflavone 7-*O*-glucosyltransferase) showed a perfect negative correlation across all the stages and genotypes studied (**Figures 5**, **6**). The expression level of *Gma*-miRNA26 in NRC7 and NRC37 genotype was in the following order: 35 DAF > 45 DAF > 55 DAF > 65 DAF while *vice-versa* in *Gma*-miRNA28 (**Figures 5**, **6**). In addition, the expression of both the target genes, i.e., *Glyma.10G197900* (4-coumarate-CoA ligase) and *Glyma.09G127200* (isoflavone 7-*O*-glucosyltransferase) were in opposite order to their corresponding miRNAs in both the genotypes at all the stages. Although the trend of increasing target accumulation in both the genotypes was same, but the total net accumulation was significantly higher in NRC37 compared to NRC7. The enzyme 4-coumarate: CoA ligase (4CL; EC 6.2.1.12) is required for the biosynthesis of a diverse array of plant natural phenylpropanoid products including isoflavones (Hahlbrock and Scheel, 1989; Whetten and Sederoff, 1995; Douglas, 1996). It catalyzes conversion of *p*-Coumarate into its CoA ester *p*-Coumaroyl CoA at the third step of isoflavone pathway and has been known since the 1960 (Russell and Conn, 1967; Russell, 1971; Knobloch and Hahlbrock, 1975). Being the third enzyme of the pathway, it may also play the central role in regulating the overall flux of the hydroxycinnamic acids into downstream biosynthesis of isoflavones (Dixon and Paiva, 1995). As a result of decreased accumulation of *Gma*-miRNA26 in decreasing order (35 DAF > 45 DAF > 55 DAF > 65 DAF), higher expression of 4-coumarate-CoA ligase in increasing order (35 DAF < 45 DAF < 55 DAF < 65 DAF) could potentially help moving flux more rapidly leading to increased accumulation of total isoflavone in NRC37 compared to NRC7.

In addition, isoflavone 7-*O*-glucosyltransferase in crucial enzyme of the pathway which catalyzes 7-*O*-glycosylation of the different aglycone form of isoflavones, which subsequently undergoes malonylation to produce isoflavone conjugates (Barz and Welle, 1992). The result indicated that the *Gma*-miRNA28 expression is just opposite to that of *Gma*-miRNA26 in both the genotypes. Our result further indicated that the decrease in accumulation of *Glyma.09G127200* (isoflavone 7-*O*-glucosyltransferase) in decreasing order (35 DAF > 45 DAF > 55 DAF > 65 DAF) in both the genotypes might signify decreased aglycosylation leading to increased total isoflavones in NRC37 compared to NRC7 genotypes which needs further validation. In addition, the other two target genes, i.e., *Glyma.08G181000, Glyma.10G224000* (both glucosyltransferase) targeted by *Gma*-miRNA12 and *Gma*-miRNA24, respectively, showed an opposite expression pattern than isoflavone 7-*O*-glucosyltransferase at all the stages in both the genotypes. This could indicate that these genes might not be acting same as isoflavone 7-*O*-glucosyltransferase. Like isoflavone 7-*O*-glucosyltransferase, *Glyma.08G181000* at 65 DAF in both genotypes and *Glyma.10G224000* in NRC37 showed same expression pattern. This kind of expression behaviors of these two target genes might be acting same as isoflavone 7-*O*-glucosyltransferase toward total contribution of aglyconic form of isoflavones.

Very recently, in addition to negative regulatory role, reports suggested the positive regulatory role of miRNAs where they up regulate the target genes directly or indirectly in response to different cell types and conditions and in the presence of distinct cofactors (see review Orang et al., 2014). Reports suggests the similar kind of results where certain miRNAs down-regulated the expression of their target genes resulting in lower synthesis of metabolites, while some were found to activate the expression of their target genes leading to higher metabolites synthesis (Enright et al., 2004; Place et al., 2008; Ji et al., 2009; Barozai, 2012; Nunez et al., 2013). Therefore, in addition to the numerous functions in diverse biological processes, current study suggests that newly identified miRNAs in soybean might be involved in regulating the isoflavone biosynthesis. The result presented in this manuscript opens a new avenue on the regulatory role of miRNAs during isoflavone biosynthesis in soybean which after further investigation using reverse genetics would help design better strategies for soybean engineering.

## Author Contributions

OG and AD conceived and designed the experiments. OG, DN, and SK conducted the experiment. OG, VT, AS, and SP analyzed data and wrote the paper. All authors read and approved the manuscript.

## Conflict of Interest Statement

The authors declare that the research was conducted in the absence of any commercial or financial relationships that could be construed as a potential conflict of interest.

## Footnotes

1 http://www.mirbase.org

2 http://www.mirbase.org

3 http://www.ncbi.nlm.nih.gov/UniGene/lbrowse2.cgi?TAXID=3847

4 www.perl.org

5 www.ncbi.nlm.nih.gov/VecScreen/VecScreen.html

6 www.repeatmasker.org/

7 www.clustal.org/

8 http://www.plantgdb.org/XGDB/phplib/download.php?GDB=Gm

9 http://plantgrn.noble.org/psRNATarget/

10 http://bioinfo.cau.edu.cn/agriGO/

11 www.genome.jp/kegg

## Supplementary Material

FIGURE S1 **(A)** Developmental stages of soybean embryos during the study period; **(B)** PCR based validation of primers of five newly identified miRNAs **(C)** and their corresponding target genes involved in isoflavone biosynthetic pathway. Agarose gel showing PCR products. Three reference genes for both miRNAs (5s rRNA, Gma-miR172ab and Gma-miR1520d) and target genes (EF 1α 2a, cyclophillin and actin 2/7) were taken for data normalization. NTC represents non-template control. DAF-days after flowering; M-100 bp marker.

FIGURE S2 **HPLC chromatogram showing the separation of three aglyconic forms (daidzein, glycitein, and genistein) of isoflavones in Indian soybean**.

FIGURE S3 **Stable secondary structures of top five new isoflavone biosynthetic pathway related miRNAs of Glycine max**. Mature miRNAs are highlighted with black line.

FIGURE S4 **Sequence alignment and phylogenetic analysis of the new miRNAs; (A)** *Gma*-miRNA12; **(B)** *Gma*-miRNA26; **(C)** *Gma*-miRNA28; **(D)** Gma-miRNA29.

TABLE S1 **List of new miRNA primers used in this study**.

TABLE S2 **List of primers of target transcripts used in this study**.

TABLE S3 **Isoflavone profiling of two contrasting soybean genotypes at four seed developmental stages**.

FILE S1 **List of new miRNAs**.

FILE S2 **Target transcripts**.

FILE S3 **GO and KEGG**.

## References

[B1] Aerenhouts D. Hebbelinck M. De Vriese S. Clarys P. (2010). Soy consumption fits within a healthy lifestyle. *Nutr. Food Sci.* 40 362–370. 10.1108/00346651011062005

[B2] Akashi T. Aoki T. Ayabe S. (1999). Cloning and functional expression of a cytochrome P450 cDNA encoding 2-hydroxyisofl avanone synthase involved in biosynthesis of the isoflavonoid skeleton in licorice. *Plant Physiol.* 121 821–828. 10.1104/pp.121.3.821 10557230 PMC59444

[B3] Akter A. Islam M. M. Mondal S. I. Mahmud Z. Jewel N. A. Ferdous S. (2014). Computational identification of miRNA and targets from expressed sequence tags of coffee (*Coffea arabica*). *Saudi J. Biol. Sci.* 21 3–12. 10.1016/j.sjbs.2013.04.007 24596494 PMC3937464

[B4] Barozai M. Y. K. (2012). Identification and characterization of the microRNAs and their targets in *Salmo salar*. *Gene* 499 163–168. 10.1016/j.gene.2012.03.006 22425976

[B5] Barz W. Welle R. (1992). “Biosynthesis and metabolism of isoflavones and pterocarpan phytoalexins in chickpea, soybean and phytopathogenic fungi,” in *Phenolic Metabolism in Plants* Vol. 26 eds Stafford H. Ibrahim R. (New York, NY: Plenum Publishing Corp), 139–164.

[B6] Benjamini Y. Hochberg Y. (1995). Controlling the false discovery rate: a practical and powerful approach to multiple testing. *J. R. Stat. Soc. Ser. B Methodol.* 57 289–300.

[B7] Boke H. Ozhuner E. Turktas M. Parmaksiz I. Ozcan S. Unver T. (2015). Regulation of the alkaloid biosynthesis by miRNA in opium poppy. *Plant Biotechnol. J.* 13 409–420. 10.1111/pbi.12346 25735537

[B8] Bulgakov V. P. Avramenko T. V. (2015). New opportunities for the regulation of secondary metabolism in plants: focus on microRNAs. *Biotechnol. Lett.* 37 1719–1727. 10.1007/s10529-015-1863-8 26003096

[B9] Clarkson T. (2000). Soy phytoestrogens: what will be their role in postmenopausal hormone replacement therapy? *Menopause* 7 71–75. 10.1097/00042192-200007020-00002 10746888

[B10] Dai X. Zhao P. X. (2011). psRNATarget: a plant small RNA target analysis server. *Nucleic Acids Res.* 39 155–159. 10.1093/nar/gkr319 21622958 PMC3125753

[B11] Dhaubhadel S. Gijzen M. Moy P. Farhangkhoee M. (2007). Transcriptome analysis reveals a critical role of *CHS7* and *CHS8* genes for isoflavone synthesis in soybean seeds. *Plant Physiol.* 143 326–338. 10.1104/pp.106.086306 17098860 PMC1761968

[B12] Dhaubhadel S. McGarvey B. D. Williams R. Gijzen M. (2003). Isoflavonoid biosynthesis and accumulation in developing soybean seeds. *Plant Mol. Biol.* 53 733–743. 10.1023/B:PLAN.0000023666.30358.ae 15082922

[B13] Dixon R. A. Paiva N. L. (1995). Stress-induced phenylpropanoid metabolism. *Plant Cell* 7 1085–1097. 10.1105/tpc.7.7.1085 12242399 PMC160915

[B14] Douglas C. J. (1996). Phenylpropanoid metabolism and lignin biosynthesis, from weeds to trees. *Trends Plant Sci.* 1 171–178. 10.1016/1360-1385(96)10019-4

[B15] Du Z. Zhou X. Ling Y. Zhang Z. Su Z. (2010). agriGO: a GO analysis toolkit for the agricultural community. *Nucleic Acids Res.* 38 64–70. 10.1093/nar/gkq310 PMC2896167 20435677

[B16] Eldem V. Akcay U. C. Ozhuner E. Bakir Y. Uranbey S. Unver T. (2012). Genome-wide identification of miRNAs responsive to drought in peach (*Prunus persica*) by high-throughput deep sequencing. *PLoS ONE* 7:e50298 10.1371/journal.pone.0050298 PMC3515591 23227166

[B17] Enright A. J. Bino J. Gaul U. Tuschl T. Sander C. Marks D. S. (2004). MicroRNA targets in Drosophila. *Genome Biol.* 5 R1. 10.1186/gb-2003-5-1-r1 PMC395733 14709173

[B18] Fang X. Zhao Y. Ma Q. Huang Y. Wang P. Zhang J. (2013). Identification and comparative analysis of cadmium tolerance-associated miRNAs and their targets in two soybean genotypes. *PLoS ONE* 8:e81471 10.1371/journal.pone.0081471 PMC3867309 24363811

[B19] FAO (2013). Available at. http://www.fao.org/docrep/t0532e/t0532e02.htm

[B20] Gou J. Y. Felippes F. F. Liu C. J. Weigel D. Wang J. W. (2011). Negative regulation of anthocyanin biosynthesis in Arabidopsis by a miR156-targeted SPL transcription factor. *Plant Cell* 23 1512–1522. 10.1105/tpc.111.084525 21487097 PMC3101539

[B21] Griffiths-Jones S. Moxon S. Marshall M. Khanna A. Eddy S. R. Bateman A. (2005). Rfam: annotating non-coding RNAs in complete genomes. *Nucleic Acids Res.* 33 121–124. 10.1093/nar/gki081 PMC540035 15608160

[B22] Guo N. Ye W. Yan Q. Huang J. Wu Y. Shen D. (2014). Computational identification of novel microRNAs and targets in *Glycine max*. *Mol. Biol. Rep.* 41 4965–4975. 10.1007/s11033-014-3362-8 24728567

[B23] Gupta O. P. Karkute S. G. Banerjee S. Meena N. L. Dahuja A. (2017). Contemporary understanding of miRNA-based regulation of secondary metabolites biosynthesis in plants. *Front. Plant Sci.* 8:374 10.3389/fpls.2017.00374 PMC5372812 28424705

[B24] Gupta O. P. Permar V. Koundal V. Singh U. D. Praveen S. (2012). MicroRNA regulated defense responses in *Triticum aestivum* L. during *Puccinia graminis* f.sp. *tritici* infection. *Mol. Biol. Rep.* 39 817–822. 10.1007/s11033-011-0803-5 21633895

[B25] Gupta O. P. Sharma P. Gupta R. K. Sharma I. (2014a). Current status on role of miRNAs during plant fungus interaction. *Physiol. Mol. Plant Pathol.* 85 1–7. 10.1016/j.pmpp.2013.10.002

[B26] Gupta O. P. Sharma P. Gupta R. K. Sharma I. (2014b). MicroRNA mediated regulation of metal toxicity in plants: present status and future perspectives. *Plant Mol. Biol.* 84 1–18. 10.1007/s11103-013-0120-6 23975146

[B27] Gurkok T. Turktas M. Parmaksiz I. Unver T. (2014). Transcriptome profiling of alkaloid biosynthesis in elicitor induced opium poppy. *Plant Mol. Biol. Rep.* 33 673–688. 10.1007/s11105-014-0772-7

[B28] Hahlbrock K. Scheel D. (1989). Physiology and molecular biology of phenylpropanoid metabolism. *Annu. Rev. Plant Physiol. Plant Mol. Biol.* 40 347–369. 10.1146/annurev.pp.40.060189.002023

[B29] Hao D. C. Yang L. Xiao P. G. Liu M. (2012). Identification of Taxus microRNAs and their targets with high-throughput sequencing and degradome analysis. *Physiol. Plant.* 146 388–403. 10.1111/j.1399-3054.2012.01668.x 22708792

[B30] Henkel J. (2000). Soy: health claims for soy protein, question about other components. *FDA Consum.* 34 18–20. 11521249

[B31] Ji J. Shi J. Budhu A. Yu Z. Forgues M. Roessler S. (2009). MicroRNA expression, survival, and response to interferon in liver cancer. *N. Engl. J. Med.* 361 1437–1447. 10.1056/NEJMoa0901282 19812400 PMC2786938

[B32] Jung W. Yu O. Lau S. C. O’Keefe D. P. Odell J. Fader G. (2000). Identification and expression of isoflavone synthase, the key enzyme for biosynthesis of isoflavones in legumes. *Nat. Biotechnol.* 18 208–212. 10.1038/72671 10657130

[B33] Khraiwesh B. Arif M. A. Seumel G. I. Ossowski S. Weigel D. Reski R. (2010). Transcriptional control of gene expression by microRNAs. *Cell* 140 111–122. 10.1016/j.cell.2009.12.023 20085706

[B34] Knobloch K. H. Hahlbrock K. (1975). Isoenzyme der *p-Cumarat*: CoA ligase aus zellsuspensionskulturen von *glycine max*. *Planta Med.* 28 102–106. 10.1055/s-0028-1104769 1237910

[B35] Kulcheski F. R. de Oliveira L. F. V. Molina L. G. Almerao M. P. Rodrigues F. A. Marcolino J. (2011). Identification of novel soybean microRNAs involved in abiotic and biotic stresses. *BMC Genomics* 12:307 10.1186/1471-2164-12-307 PMC3141666 21663675

[B36] Kumar V. Rani A. Dixit A. K. Pratap D. Bhatnagar D. (2010). A comparative assessment of total phenolic content, ferric reducing-anti-oxidative power, free radical-scavenging activity, vitamin C and isoflavone content in soybean with varying seed coat colour. *Food Res. Int.* 43 323–328. 10.1016/j.foodres.2009.10.019

[B37] Li X. Wang X. Zhang S. Liu D. Duan Y. Dong W. (2012). Identification of soybean microRNAs involved in soybean cyst nematode infection by deep sequencing. *PLoS ONE* 7:e39650 10.1371/journal.pone.0039650 PMC3384596 22802924

[B38] Limer J. L. Speirs V. (2004). Phyto-oestrogens and breast cancer chemoprevention. *Breast Cancer Res.* 6 119–127. 10.1186/bcr781 15084232 PMC400678

[B39] Liu Q. Chen Y. Q. (2010). A new mechanism in plant engineering: the potential roles of microRNAs in molecular breeding for crop improvement. *Biotechnol. Adv.* 28 301–307. 10.1016/j.biotechadv.2010.01.002 20067828

[B40] Livak K. J. Schmittgen T. D. (2001). Analysis of relative gene expression data using real-time quantitative PCR and the DDCT method. *Methods* 25 402–408. 10.1006/meth.2001.1262 11846609

[B41] Lu Y. Yang X. (2010). Computational Identification of Novel MicroRNAs and their targets in Vigna unguiculata. *Comp. Funct. Genomics* 2010:128297 10.1155/2010/128297 PMC2929582 20811611

[B42] Navarro L. Dunoyer P. Jay F. Arnold B. Dharmasiri N. Estelle M. (2006). A plant miRNA contributes to antibacterial resistance by repressing auxin signaling. *Science* 312 436–439. 10.1126/science.1126088 16627744

[B43] Ndakidemi P. A. Dakora F. D. (2003). Legume seed flavonoids and nitrogenous metabolites as signals and protectants in early seedling development. *Rev. Funct. Plant Biol.* 30 729–745. 10.1071/FP03042 32689057

[B44] Ng D. W. Zhang C. Miller M. Palmer G. Whiteley M. Tholl D. (2011). *Cis*- and *trans*-regulation of miR163 and target genes confers natural variation of secondary metabolites in two *Arabidopsis* species and their allopolyploids. *Plant Cell* 23 1729–1740. 10.1105/tpc.111.083915 21602291 PMC3123960

[B45] Nigam D. Kadimi P. K. Kumar S. Mishra D. C. Rai A. (2015). Computational analysis of miRNA-target community network reveals cross talk among different metabolisms. *Genom. Data* 5 292–296. 10.1016/j.gdata.2015.04.028 26484271 PMC4584007

[B46] Nunez Y. O. Truitt J. M. Gorini G. Ponomareva O. N. Blednov Y. A. Harris R. A. (2013). Positively correlated miRNA-mRNA regulatory networks in mouse frontal cortex during early stages of alcohol dependence. *BMC Genomics* 14:725 10.1186/1471-2164-14-725 PMC3924350 24148570

[B47] Oh T. J. Wartell R. M. Cairney J. Pullman G. S. (2008). Evidence for stage-specific modulation of specific microRNAs (miRNAs) and miRNA processing components in zygotic embryo and female gametophyte of loblolly pine (*Pinus taeda*). *New Phytol.* 179 67–80. 10.1111/j.1469-8137.2008.02448.x 18433430

[B48] Orang A. V. Safaralizadeh R. Kazemzadeh-Bavili M. (2014). Mechanisms of miRNA-mediated gene regulation from common down regulation to mRNA-specific up regulation. *Int. J. Genomics* 2014 1–15. 10.1155/2014/970607 PMC4142390 25180174

[B49] Pandey B. Gupta O. P. Pandey D. M. Sharma I. Sharma P. (2013). Identification of new stress-induced microRNA and their targets in wheat using computational approach. *Plant Signal. Behav.* 8:e23932 10.4161/psb.23932 PMC3906146 23511197

[B50] Pani A. Mahapatra R. K. (2013). Computational identification of microRNAs and their targets in *Catharanthus roseus* expressed sequence tags. *Genom. Data* 1 2–6. 10.1016/j.gdata.2013.06.001 26484050 PMC4608865

[B51] Place R. F. Li L. C. Pookot D. Noonan E. J. Dahiya R. (2008). MicroRNA-373 induces expression of genes with complementary promoter sequences. *Proc. Natl. Acad. Sci. U.S.A.* 105 1608–1613. 10.1073/pnas.0707594105 18227514 PMC2234192

[B52] Ralston L. Subramanian S. Matsuno M. Yu O. (2005). Partial reconstruction of flavonoid and isoflavone biosynthesis in yeast using soybean type I and type II chalcone isomerases. *Plant Physiol.* 137 1375–1388. 10.1104/pp.104.054502 15778463 PMC1088328

[B53] Riaz M. N. (2006). *Soy Applications in Food.* Boca Raton, FL: CRC Press.

[B54] Ro S. Park C. Jin J. Sanders K. M. Yan W. (2006). A PCR based method for detection and quantification of small RNAs. *Biochem. Biophys. Res. Commun.* 351 756–763. 10.1016/j.bbrc.2006.10.105 17084816 PMC1934510

[B55] Rochfort S. Panozzo J. (2007). Phytochemicals for health, the role of pulses. *Agric. Food Chem.* 55 7981–7994. 10.1021/jf071704w 17784726

[B56] Rose D. Boyar A. Wynder E. (1986). International comparisons of mortality rates for cancer of the breast, ovary, prostate and colon, and per capita food consumption. *Cancer* 58 2363–2371. 10.1002/1097-0142(19861201)58:11<2363::AID-CNCR2820581102>3.0.CO;2-# 3768832

[B57] Russell D. W. (1971). The metabolism of aromatic compounds in higer plants. X. Properties of the cinnamic acid 4-hydroxylase of pea seedlings and some aspects of its metabolic and developmental control. *J. Biol. Chem.* 246 3870–3878. 4397825

[B58] Russell D. W. Conn E. E. (1967). The cinnamic acid 4-hydroxylase of pea seedlings. *Arch. Biochem. Biophys.* 122 256–258. 10.1016/0003-9861(67)90150-6 4383827

[B59] Schroder J. (2000). “The chalcone/stilbene synthase-type family of condensing enzymes,” in *Comprehensive Natural Product Chemistry. Polyketide and Other Secondary Metabolites Including Secondary Metabolites Including Fatty Acids and Their Derivatives*, ed. Sankawa U. (Oxford: Elsevier), 749–771.

[B60] Setchell K. D. Brown N. M. Desai P. B. Zimmer-Nechimias L. Wolfe B. Jakate A. S. (2003). Bioavailability, disposition, and dose-response effects of soy isoflavones when consumed by healthy women at physiologically typical dietary intakes. *J. Nutr.* 133 1027–1035. 12672914 10.1093/jn/133.4.1027

[B61] Song Q. X. Liu Y. F. Hu X. Y. Zhang W. K. Ma B. Chen S. Y. (2011). Identification of miRNAs and their target genes in developing soybean seeds by deep sequencing. *BMC Plant Biol.* 11:5 10.1186/1471-2229-11-5 PMC3023735 21219599

[B62] Turner D. H. Mathews D. H. (2009). NNDB: the nearest neighbor parameter database for predicting stability of nucleic acid secondary structure. *Nucleic Acids Res.* 38 280–282. 10.1093/nar/gkp892 PMC2808915 19880381

[B63] Turner M. Yu O. Subramanian S. (2012). Genome organization and characteristics of soybean microRNAs. *BMC Genomics* 13:169 10.1186/1471-2164-13-169 PMC3481472 22559273

[B64] Tuteja J. H. Zabala G. Varala K. Hudson M. Vodkin L. O. (2009). Endogenous, tissue-specific short interfering RNAs silence the chalcone synthase gene family in *Glycine max* seed coats. *Plant Cell* 21 3063–3077. 10.1105/tpc.109.069856 19820189 PMC2782299

[B65] Vashisht I. Mishra P. Pal T. Chanumolu S. Singh T. R. Chauhan R. S. (2015). Mining NGS transcriptomes for miRNAs and dissecting their role in regulating growth, development, and secondary metabolites production in different organs of a medicinal herb *Picrorhiza kurroa*. *Planta* 241 1255–1268. 10.1007/s00425-015-2255-y 25663583

[B66] Vyn T. J. Yin X. Bruulsema T. W. Jackson J. C. Rajcan I. Bruder S. M. (2002). Potassium fertilization effects on isoflavones concentrations in soybean. *J. Agric. Food Chem.* 50 3501–3506. 10.1021/jf0200671 12033818

[B67] Wang H. Murphy P. A. (1994). Isoflavone content in commercial soybean foods. *J. Agric. Food Chem.* 42 1666–1673. 10.1021/jf00044a016

[B68] Whetten R. Sederoff R. (1995). Lignin biosynthesis. *Plant Cell* 7 1001–1013. 10.1105/tpc.7.7.1001 12242395 PMC160901

[B69] Xu F. Liu Q. Chen L. Kuang J. Walk T. Wang J. (2013). Genome-wide identification of soybean microRNAs and their targets reveals their organ-specificity and responses to phosphate starvation. *BMC Genomics* 14:66 10.1186/1471-2164-14-66 PMC3673897 23368765

[B70] Xue C. Li F. He T. Liu G. P. Li Y. Zhang X. (2005). Classification of real and pseudo microRNA precursors using local structure-sequence features and support vector machine. *BMC Bioinformatics* 6:310 10.1186/1471-2105-6-310 PMC1360673 16381612

[B71] Yanik H. Turktas M. Dundar E. Hernandez P. Dorado G. Unver T. (2013). Genome-wide identification of alternate bearing-associated microRNAs (miRNAs) in olive (*Olea europaea* L.). *BMC Plant Biol.* 13:10 10.1186/1471-2229-13-10 PMC3564680 23320600

[B72] Ye C. Y. Xu H. Shen E. Liu Y. Wang Y. Shen Y. (2014). Genome wide identification of non-coding RNAs interacted with miRNAs in soybean. *Front. Plant Sci.* 5:743 10.3389/fpls.2014.00743 PMC4274897 25566308

[B73] Zeng Q. Y. Yang C. Y. Ma Q. B. Li X. P. Dong W. W. Zeng H. N. (2012). Identification of wild soybean miRNAs and their target genes responsive to aluminum stress. *BMC Plant Biol.* 12:182 10.1186/1471-2229-12-182 PMC3519564 23040172

[B74] Zhang B. Pan X. Cannon C. H. Cobb G. P. Anderson T. A. (2006). Conservation and divergence of plant microRNA genes. *Plant J.* 46 243–259. 10.1111/j.1365-313X.2006.02697.x 16623887

[B75] Zhang B. Wang Q. (2015). MicroRNA-based biotechnology for plant improvement. *J. Cell. Physiol.* 230 1–15. 10.1002/jcp.24685 24909308

[B76] Zhang B. H. Ping P. X. Lian W. Q. Cobb G. P. Anderson T. A. (2005). Identification and characterization of new plant microRNAs using EST analysis. *Cell Res.* 15 336–360. 10.1038/sj.cr.7290302 15916721

[B77] Zhang S. Wang Y. Li K. Zou Y. Chen L. Li X. (2014). Identification of cold-responsive miRNAs and their target genes in nitrogen-fixing nodules of soybean. *Int. J. Mol. Sci.* 15 13596–13614. 10.3390/ijms150813596 25100171 PMC4159813

