# Supplementary figures and images for: Regulation of Isoflavone Biosynthesis by miRNAs in Two Contrasting Soybean Genotypes at Different Seed Developmental Stages
Source: Front Plant Sci. 2017 Apr 13;8:567. doi: 10.3389/fpls.2017.00567 (PMC5390031; doi:10.3389/fpls.2017.00567)

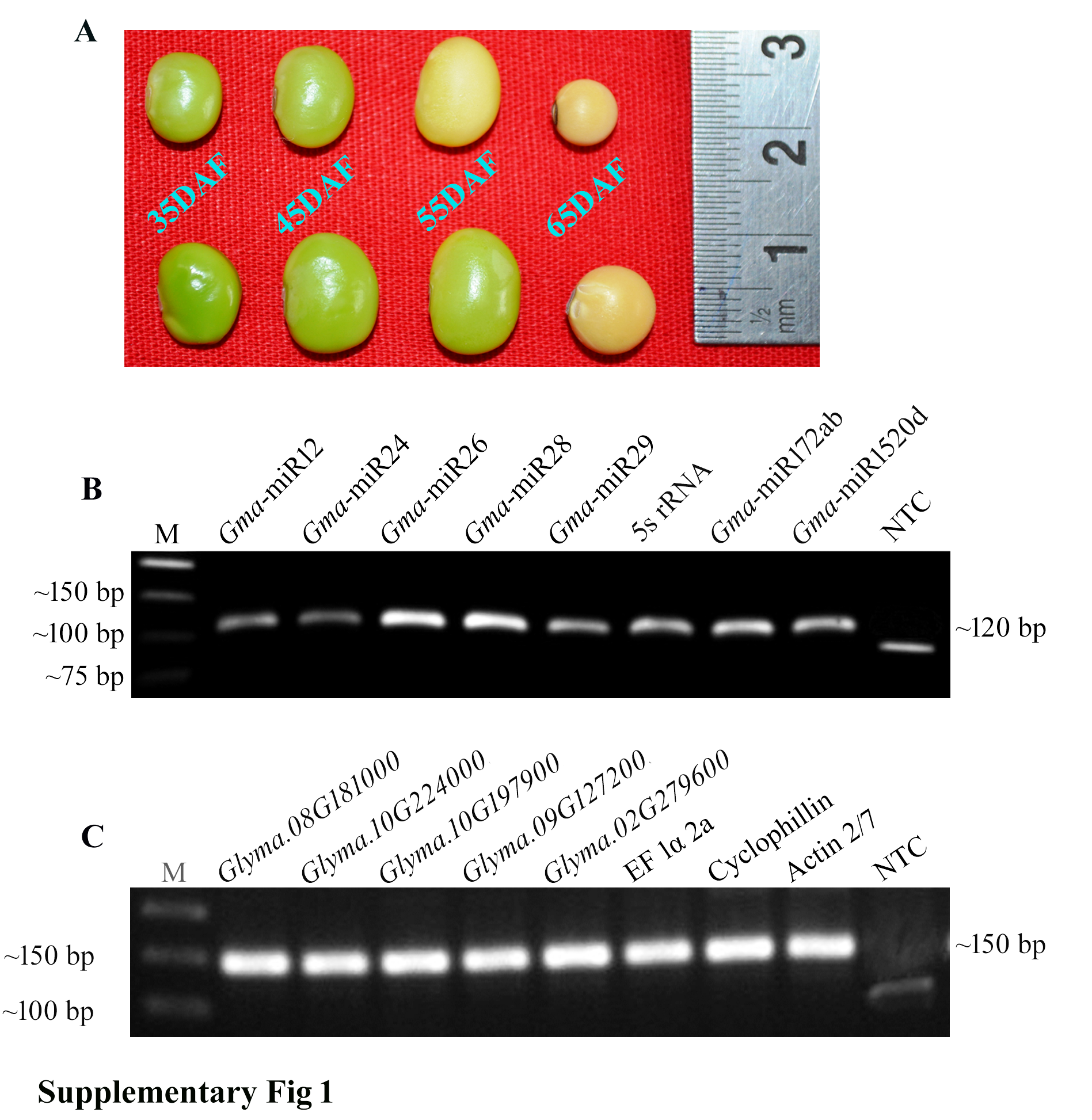

Supplement: FIGURE S1 [file Image_1.TIF]

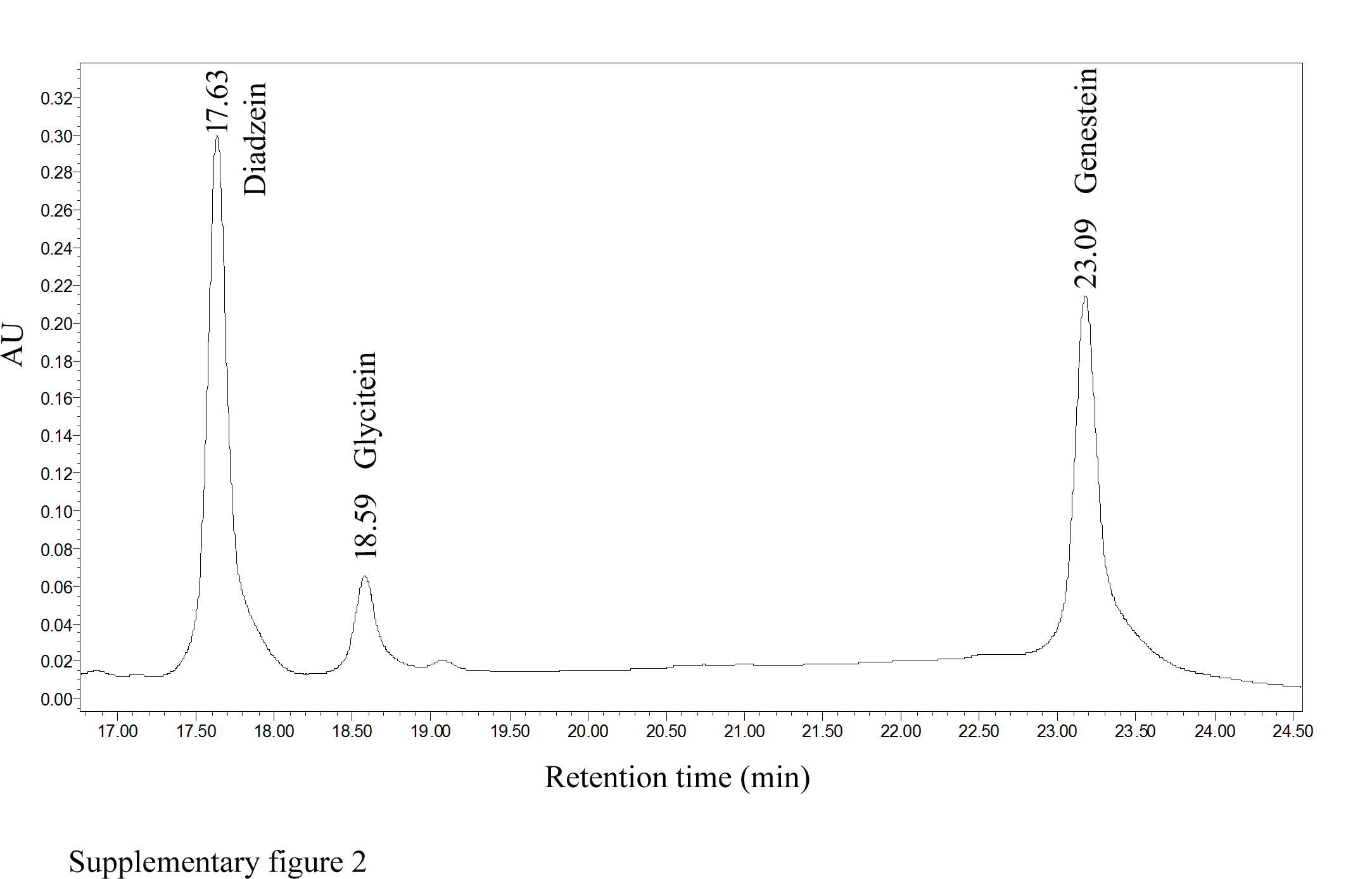

Supplement: FIGURE S2 [file Image_2.TIF]

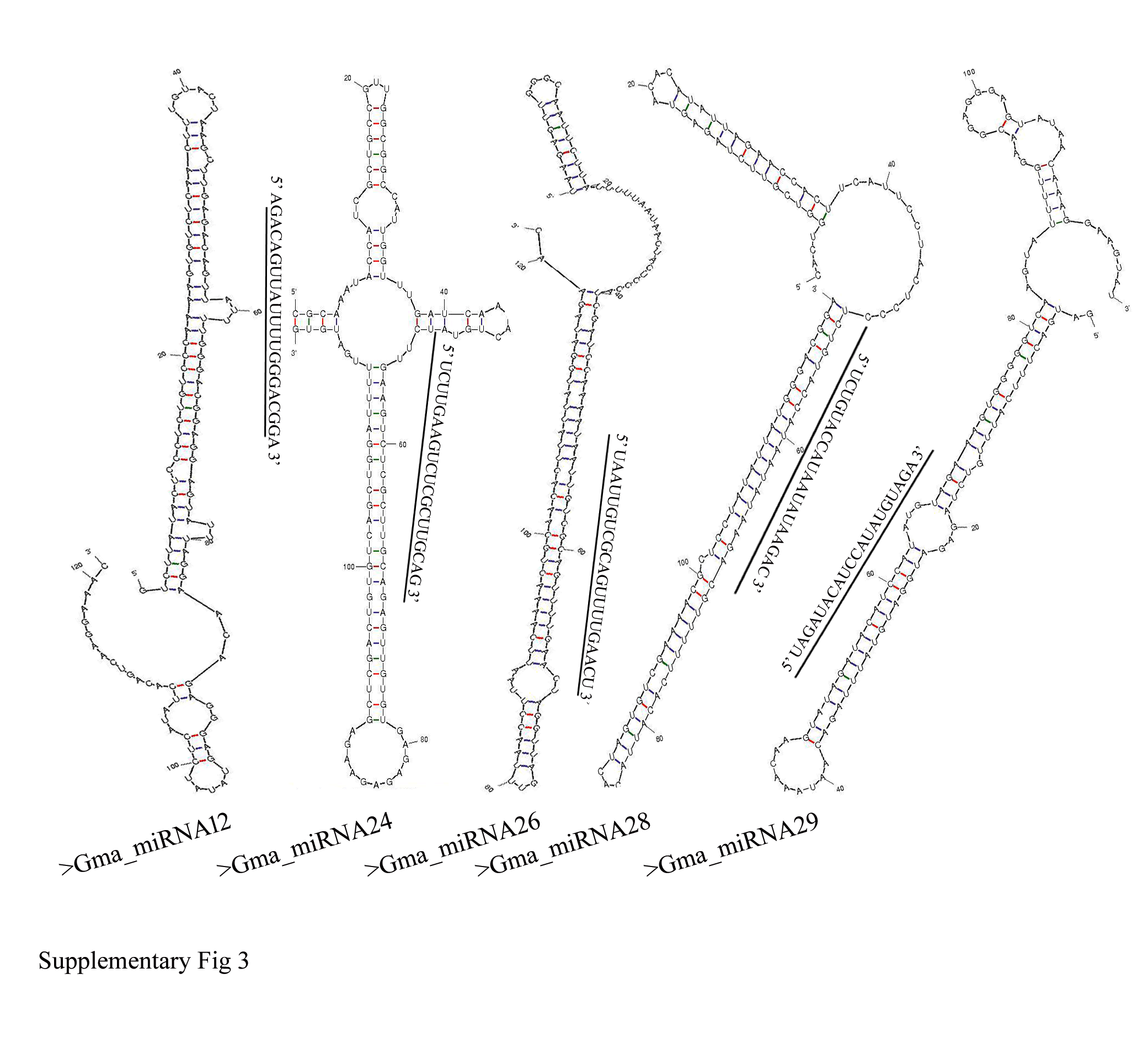

Supplement: FIGURE S3 [file Image_3.TIF]

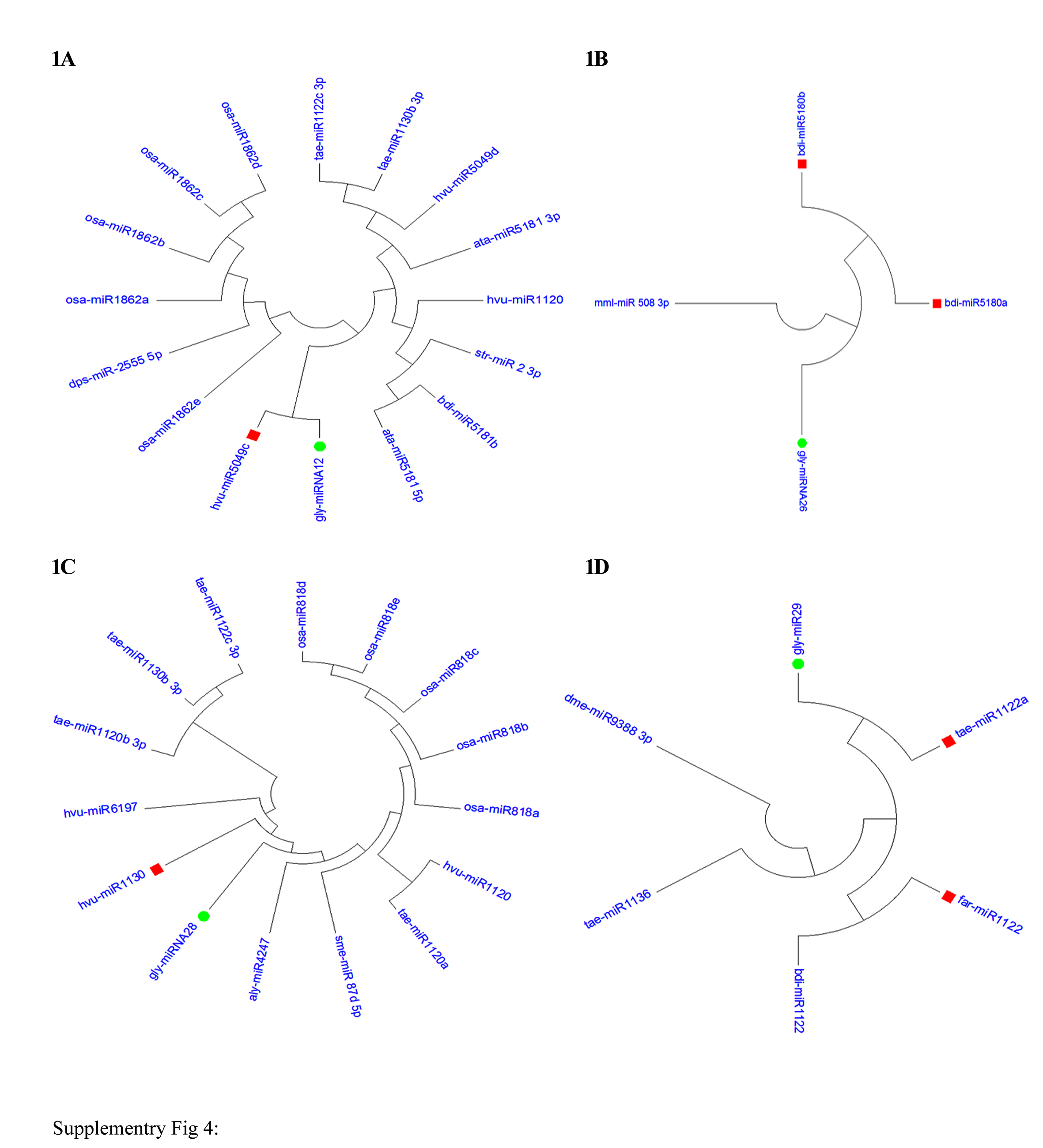

Supplement: FIGURE S4 [file Image_4.TIF]
