# Supplementary material for: Regulation of Isoflavone Biosynthesis by miRNAs in Two Contrasting Soybean Genotypes at Different Seed Developmental Stages
Source: Front Plant Sci. 2017 Apr 13;8:567. doi: 10.3389/fpls.2017.00567 (PMC5390031; doi:10.3389/fpls.2017.00567)
Supplement: TABLE S1 [file Table_1.DOC]

Supplementary table 1:

| **Sr. No.** | **Name** | **Mature miRNA sequence** | **Primer (5’ to 3’)** | **Tm (°C)** |
| --- | --- | --- | --- | --- |
| 1. | RTQ primer | - | CGA ATT CTA GAG CTC GAG GCA GGC GAC ATG GCT GGC TAG TTA AGC TTG GTA CCG AGC TCG GAT CCA CTA GTC C(T)25 | 75-76 |
| 2. | RTQ-UNIr | - | CGA ATT CTA GAG CTC GAG GCA GG | 72 |
| 3. | >Gma_miRNA12 | AGA CAG UUA UUU UGG GAC GGA | AGA CAG TTA TTT TGG GAC GGA | 60 |
| 4. | >Gma_miRNA24 | UCU UGA AGU CUC GCU UGC AG | TCT TGA AGT CTC GCT TGC AG | 60 |
| 5. | >Gma_miRNA26 | UAA UUG UCG CAG UUU UGA ACU | TAA TTG TCG CAG TTT TGA ACT | 56 |
| 6. | >Gma_miRNA28 | UCU GUA CCA UAA UAU AAG AC | TCT GTA CCA TAA TAT AAG AC | 52 |
| 7. | >Gma_miRNA29 | UAG AUA CAU CCA UAU GUA GA | TAG ATA CAT CCA TAT GTA GA | 52 |
| **Internal control genes** | | | | |
| 8. | 5s rRNA | - | GGA AAA ATA GCT CGG CGC CA | 64 |
| 9. | miR172ab | AGAAUCUUGAUGAUGCUGCAU | AGA ATC TTG ATG ATG CTG CA | 60 |
| 10. | miR1520d | AUCAGAACAUGACACGUGACAA | TCA GAA CAT GAC ACG TGA CAA | 60 |
