# Supplementary material for: Regulation of Isoflavone Biosynthesis by miRNAs in Two Contrasting Soybean Genotypes at Different Seed Developmental Stages
Source: Front Plant Sci. 2017 Apr 13;8:567. doi: 10.3389/fpls.2017.00567 (PMC5390031; doi:10.3389/fpls.2017.00567)
Supplement: TABLE S2 [file Table_2.DOC]

Supplementary table 2:

| Sr. No. | miRNA | miR binding site on target (bp) | | Target | Target total length (bp) | Primer Name | Primer region (bp) | F Primer (5’ to 3’) | | R Primer (5’ to 3’) | Amplicon size (bp) | Tm (^0^C)  (PrimerBlast) | |
| --- | --- | --- | --- | --- | --- | --- | --- | --- | --- | --- | --- | --- | --- |
| 1. | >Gma_miRNA12 | 429 | | Glyma.08G181000.1 | 1802 | RA-5’ | 210-354 | CAT GCT CCC ATG GCT GGC | | TGG TTC TAA GTG TTT CGG GGT T | 145 | 60 | |
|  |  |  |  |  |  | RA-Mid | 403-545 | GAA GGC GCA GAG AGC ACA AT | | CAA GCA GCT GCG AAG TCA TAC | 143 | 59 | |
|  |  |  |  |  |  | RA-3’ | 644-784 | TGA AGG ATT ACT CGC TCG CA | | CAA AAG AAG CCC TTT CAC CTG T | 141 | 59 | |
| 2. | > Gma _miRNA24 | 563 | | Glyma.10G224000.1 | 3117 | RA-5’ | 153-298 | GAA CCA GTC GTT GCC GC | | GGA ATT CCC TCT TTC CTG TCA T | 146 | 59 | |
|  |  |  |  |  |  | RA-Mid | 450-590 | CGA GTC AAC AGT GAG TGG AA | | TGA CCA TAC TCC TGA AGT CTC CT | 141 | 59 | |
|  |  |  |  |  |  | RA-3’ | 664-804 | GGT GGC GAT CCT CGT GTA TT | | ATC AGG TGC TGT GCA TGC TG | 141 | 60 | |
| 3. | > Gma _miRNA26 | 1244 | | Glyma.10G197900.1 | 1713 | RA-5’ | 838-987 | GGT ATT ACA ACA GTT TTG ATG CAG A | | GCT GGA CAA GTC ACA CTT AAC | 150 | 58 | |
|  |  |  |  |  |  | RA-Mid | 1196-1337 | ACA TTG AAA CGG GGA AGC CT | | TCA CCA GTC CTC AAC CAA CC | 142 | 59 | |
|  |  |  |  |  |  | RA-3’ | 1405-1544 | AAT GGG TAT CAG GTG GCT CC | | GAA CCT GCT GCTATC ACC ACA | 140 | 59 | |
| 4. | > Gma _miRNA28 | 1690 | | Glyma.09G127200.1 | 1770 | RA-5’ | 1265-1404 | GAG AGA ACG AAG GGG AGG GG | | ATT GGC ACC CCT TCA CAC AC | 140 | 60 | |
|  |  |  |  |  |  | RA-Mid | 1578-1722 | GGG TTT TTG AGA TGA AAA TAG GTG C | | CAT GGG GTT AAG CTC TGT ACC A | 145 | 59 | |
|  |  |  |  |  |  | RA-3’ | 1625-1769 | GGG TCT TCA CTT GTT GCG TTC | | AGA ACT AAA ACA TGT GGC GTA CAA | 145 | 59 | |
| 5. | > Gma _miRNA29 | 1367 | | Glyma.02G279600.1 | 1992 | RA-5’ | 425-568 | GTC TTC CGC ATT TGG GGT G | | CAC CAT AAG CAG CAA AAG CAG T | 144 | 59 | |
|  |  |  |  |  |  | RA-Mid | 1321-1470 | TCA GCC ACC TTG GGA CTT AAA | | TTG GGT GGA GGA CCC AAA AG | 150 | 59 | |
|  |  |  |  |  |  | RA-3’ | 1572-1715 | ACA AAA GCT GAG GGA ATG ATG A | | TCT ATT TTC ATA GAA AGC ACA AGC A | 144 | 57 | |
| **Internal control genes** | | | | | | | | | | | | | |
| **Sr. No.** | **Gene** | | **F Primer (5’ to 3’)** | | | | | | **R Primer (5’ to 3’)** | | **Amplicon size (bp)** | | **Tm (^0^C)** |
| 1. | EF1α2a | | GCT CTC ACA GAG GCT CTT CCC | | | | | | ATG ATG ATA ACC TGG GCA GTG A | | 147 | | 59 |
| 2. | Cyclophilin | | GCA CGA GTC CTC TTC CTC AG | | | | | | CTC GGA GTC ACG TCG GC | | 144 | | 59 |
| 3. | Actin2/7 | | ACT TGC CCA TCA GGA AGC TC | | | | | | TGT TCA CCA CCT CTG CCA AG | | 145 | | 60 |
