# Supplementary material for: Regulation of Isoflavone Biosynthesis by miRNAs in Two Contrasting Soybean Genotypes at Different Seed Developmental Stages
Source: Front Plant Sci. 2017 Apr 13;8:567. doi: 10.3389/fpls.2017.00567 (PMC5390031; doi:10.3389/fpls.2017.00567)
Supplement: TABLE S3 [file Table_3.DOC]

**Supplementary table 3:**

| **Variety** | **Seed stages** | **Daidzein (µg/gm)** | **Genistein (µg/gm)** | **Glycitein (µg/gm)** | **Total isoflavones (µg/gm)** | **Genistein: daidzein ratio** |
| --- | --- | --- | --- | --- | --- | --- |
| NRC7 | 35DAP | 43.7832±7 | 224.7944±16 | 81.8775±27 | 350.4553±50 | 5.14 |
|  | 45DAP | 177.4947±27 | 211.1679±10 | 110.8016±7 | 499.4644±44 | 1.19 |
|  | 55DAP | 269.6209±30 | 150.4749±18 | 84.4173±19 | 504.5133±67 | 0.55 |
|  | 65DAP | 200.0035±11 | 308.0631±9 | 98.2956±17 | 606.3623±37 | 1.54 |
| NRC37 | 35DAP | 431.3421±89 | 220.9328±76 | 186.9925±14 | 839.2665±181 | 0.51 |
|  | 45DAP | 597.9169±104 | 323.5381±23 | 182.4365±11 | 1103.8914±141 | 0.54 |
|  | 55DAP | 785.7509±29 | 244.7168±11 | 251.3598±18 | 1281.8275±58 | 0.34 |
|  | 65DAP | 704.0697±34 | 415.0330±4 | 263.4108±12 | 1382.5136±50 | 0.58 |

Values given are mean± standard deviation of triplicate samples.
